# Cognitive training of mice attenuates age-related decline in associative learning and behavioral flexibility

**DOI:** 10.3389/fnbeh.2024.1326501

**Published:** 2024-03-14

**Authors:** Dalia Attalla, Alexej Schatz, Katharina Stumpenhorst, York Winter

**Affiliations:** Neurobiology Department, Institute for Biology, Faculty of Life Sciences, Humboldt University of Berlin, Berlin, Germany

**Keywords:** touchscreen operant chamber, ID-based sorter, home-cage-based testing, spatial working memory, attention, reversal learning

## Abstract

Identifying factors that influence age-related cognitive decline is crucial, given its severe personal and societal impacts. However, studying aging in human or animal models is challenging due to the significant variability in aging processes among individuals. Additionally, longitudinal and cross-sectional studies often produce differing results. In this context, home-cage-based behavioral analysis over lifespans has emerged as a significant method in recent years. This study aimed to explore how prior experience affects cognitive performance in mice of various age groups (4, 12, and 22 months) using a home-cage-based touchscreen test battery. In this automated system, group-housed, ID-chipped mice primarily obtain their food during task performance throughout the day, motivated by their own initiative, without being subjected to food deprivation. Spatial working memory and attention were evaluated using the trial unique non-matching to location (TUNL) and the five-choice serial reaction time task (5-CSRTT), respectively. The same set of mice learned both of these demanding tasks. While signs of cognitive decline were already apparent in middle-aged mice, older mice exhibited poorer performance in both tasks. Mice at both 12 and 22 months displayed an increase in perseverance and a decrease in the percentage of correct responses in the TUNL test compared to the 4-month-old mice. Furthermore, during the 5-CSRTT, they exhibited higher rates of omissions and premature responses compared to their younger counterparts. Additionally, the correct response rate in 22-month-old mice was lower than that of the 4-month-old ones. However, mice that had undergone cognitive training at 4 months maintained high-performance levels when re-tested at 12 months, showing an increase in correct responses during TUNL testing compared to their untrained controls. In the 5-CSRTT, previously trained mice demonstrated higher correct response rates, fewer omissions, and reduced premature responses compared to naive control mice. Notably, even when assessed on a visual discrimination and behavioral flexibility task at 22 months, experienced mice outperformed naive 4-month-old mice. These findings highlight the advantages of early-life cognitive training and suggest that its benefits extend beyond the cognitive domains primarily targeted during early training. The success of this study was significantly aided by the fully automated home-cage-based testing system, which allows for high throughput with minimal human intervention.

## 1 Introduction

Age is a major risk factor for neurodegenerative disorders (Kern and Behl, 2009; Bilkei-Gorzo, 2014). It is associated with alterations and declines in multiple brain circuits and cognitive domains (Harada et al., 2013; Bilkei-Gorzo, 2014; Murman, 2015) but can differ remarkably between individuals (De Felice and Holland, 2018). Notably, age-related hippocampal shrinkage has been linked with deficits in executive function, working, and episodic memory, as well as processing speed (Harada et al., 2013; Bilkei-Gorzo, 2014; Murman, 2015; O’Shea et al., 2016). Age-related frontal lobe atrophy induces attention deficits, which may subsequently negatively impact memory (West, 1996; Bilkei-Gorzo, 2014). Moreover, the loss of dopamine neurons restricted glutamine uptake, and the resultant imbalance in the dopamine–glutamine relationship has been identified as a contributor to age-related cognitive decline (Hong and Rebec, 2012). Influencing these aging processes are diverse factors, ranging from individual polygenic factors to lifestyle choices. Therefore, it is essential to study the factors that underlie the enhancement or preservation of cognitive abilities during aging in order to develop better treatments (Bherer et al., 2013). For instance, previous experience has been reported to positively impact cognition by facilitating new memory acquisition and retention (Brod et al., 2013), potentially contributing to the preservation of cognitive abilities in older individuals (Gerenu et al., 2013).

Hence, studies of aging, either longitudinal or cross-sectional, are inherently complex, and the results can be contradictory. While cross-sectional studies are subject to a cohort effect, longitudinal studies, although preferred, can be distorted by practice effects as well as attrition biases (Salthouse, 2009; Brod et al., 2013; Harada et al., 2013; Wagster and King, 2019; Ritchie et al., 2020).

In a rodent model, the use of touchscreen test systems enables tasks that are analogous to the human Cambridge Neuropsychological Test Automated Battery (CANTAB) tests (Bussey et al., 1994), thus facilitating the examination of different cognitive domains, which can be assessed simply by altering cues and task protocols (Oomen et al., 2013; Remmelink et al., 2015, 2016, 2017; Rivalan et al., 2017; Kim et al., 2019; Birtalan et al., 2020; Richter, 2020; Caglayan et al., 2021a,b). However, standard touchscreen procedures for rodents often involve food restriction, handling them immediately prior to testing, and/or single housing (Horner et al., 2013; Oomen et al., 2013; Remmelink et al., 2017; Birtalan et al., 2020). In the current study, ID-chipped mice were group-housed and tested in an automated, self-paced system. This system consists of a touchscreen-equipped operant chamber connected to the home cage through an ID-based gating system. The mice collected food based on their performance and were assessed across various cognitive domains: the trial unique non-matching to location (TUNL) task assesses spatial working memory and pattern separation; the five-choice serial reaction time task (5-CSRTT) task assesses attention and impulsivity; and the visual discrimination (VD) and reversal task assesses associative learning as well as response inhibition and behavioral flexibility (Izquierdo et al., 2017; Turner et al., 2017).

Mice typically exhibited high omission rates in the 5-CSRTT task (Bari et al., 2008; Remmelink et al., 2017), an issue we addressed by modifying the functional design of the touchscreen chamber. Additionally, utilizing a combined cross-sectional and longitudinal study approach, we evaluated the impact of age and prior touchscreen training on cognitive domains.

This study was guided by the following hypotheses:

1. The innovative home-cage-based touchscreen systems are capable of detecting age-related shifts in cognitive function in TUNL and 5CSRT tasks.
2. Regular participation in cognitive tasks has an effect on age-related cognitive decline.

## 2 Materials and methods

### 2.1 Animals

Seven cohorts of female C57BL/6 J mice (Janvier Labs, France) were tested for cognitive and executive functions at different ages (Table 1). Two young cohorts (Y1 and Y2, 4 months; *n* = 7 for each cohort), one middle-aged cohort (M, 12 months; *n* = 9), one old cohort (O1, 22 months; *n* = 7), and one longitudinal cohort at the age of 4 months (L 4-mo, 4 months; *n* = 7) were tested on the TUNL and 5-CSRTT. Cohort L 4-mo was re-tested on the TUNL and 5-CSRTT at 12 months of age (L 12-mo; *n* = 7) and also trained on the VD and reversal learning tasks at 22 months (L 22-mo; *n* = 5; two mice had to be euthanized due to age-related health decline). Furthermore, one young cohort (Y3, 4 months; *n* = 8) and one old cohort (O2, 22 months; *n* = 9) were tested on the VD and reversal learning tasks for cross-sectional comparisons. Animals were kept in conventional Eurostandard type III cages under standard housing conditions (23 ± 2°C, 40%–50% humidity) with *ad libitum* access to food and water until the start of the study period.

**Table 1:** Overview of cohorts and trained tasks.

Cohort task	Y1 (*n* = 7)	Y2 (*n* = 7)	Y3 (*n* = 8)	M (*n* = 9)	O1 (*n* = 7)	O2 (*n* = 9)	L		
							4-mo (*n* = 7)	12-mo (*n* = 7)	22-mo (*n* = 5)
TUNL	✓*	✓		✓	✓		✓	✓	
5-CSRTT	✓*^#^	✓		✓	✓		✓	✓	
VD			✓			✓			✓

Y: young adult cohort; M: middle-aged cohort; O: old age cohort; L: longitudinal cohort; * Different touch screen mask position; # Different trial initiation; TUNL: trial-unique non-matching to location task; 5-CSRTT: five-choice serial reaction time task; VD: visual discrimination and reversal task.

At least 10 days prior to the study, mice from each cohort were housed together in groups of up to nine animals in a home-cage-based test system and habituated to a 10-h/14-h light/dark cycle (dark phase: 1 p.m. to 3 a.m.) to increase voluntary activity and task engagement. Animals were then weighed and habituated to the reward pellets for 3 consecutive days. Sessions were usually run between 1 p.m. and 10 a.m. the following day. Animal health assessments, additional feeding, system cleaning, and data analysis were performed between 10 a.m. and 1 p.m. Additional reward pellets (cohort Y1) or 1 g of chow (V1535-000, Ssniff, Germany; all other cohorts) were provided to each animal in the case of weight loss.

During study periods, mice could regularly access the operant chamber where they received their food in the form of reward pellets (14 mg, 5TUL Purified Rodent Tablet, TestDiet, United States). Mice carried subcutaneous radio frequency identification (RFID) chips for individual identification.

All procedures were conducted in compliance with the European Communities Council Directive 2010/63/EU and under the supervision and approval of the animal welfare officer at Humboldt University. Generally, our approach aimed to maximize welfare by using undisturbed home-cage-based experimental observation where animals did not experience damage, pain, or suffering.

### 2.2 Apparatus

**Figure 1:**
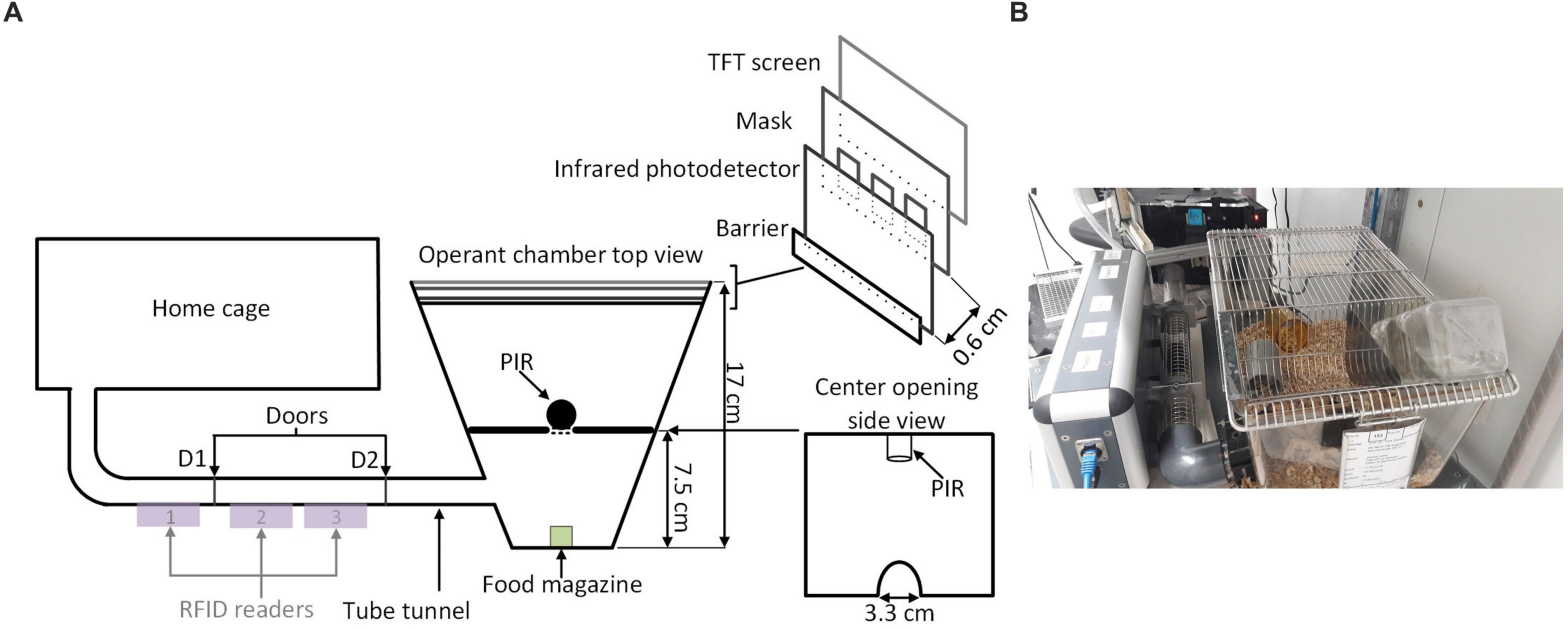
Home-cage-based touchscreen setup. **(A)** Schematic diagram; A home-cage was connected to a touchscreen operant chamber through an RFID-chip-based automated animal gating mechanism (sorter). The sorter consisted of a polycarbonate tunnel equipped with three RFID sensors and two doors (D1, D2). Its gating mechanism restricted access to the operant chamber to a single mouse. The operant chamber contained a food magazine delivering reward pellets, a loudspeaker, and a touchscreen (TS). The upmost region of the TS served as a house light. A mask and the infrared sensor grid in front of the TS defined response windows and detected nose-poke responses. For the 5-CSRTT, an additional divider with a passive infrared detector (PIR) above a central opening between the food magazine and the screen detected a mouse when it moved back from the food magazine to the screen. **(B)** A real image of the setup.

A conventional Eurostandard type III cage (43 × 27 × 18 cm) was connected through an RFID-based automated animal sorter to an operant touchscreen chamber (PhenoSys, Germany; Figure 1). Both the sorter and touchscreen chamber were controlled by PhenoSoft Control software (PhenoSys, Germany). A detailed description of the animal sorter can be found in Supplementary material 1 (Winter and Schaefers, 2011).

### 2.3 Touchscreen pre-training

Touchscreen pre-training followed previously established protocols with some modifications for the automated system (Oomen et al., 2013; Delotterie et al., 2014; Table 2). Mice first underwent a sorter habituation phase during which the sorter doors were open, allowing simultaneous chamber access to all animals for 2 h or until each mouse had visited the sorter at least three times. During this phase, animals were rewarded with 1 pellet for each head entry into the food magazine. The sorter was activated from the next phase (“Initial touch”) onward: a single animal entered the chamber, stayed within until the maximum number of trials or session duration was reached, and was subsequently sorted out before a new animal could be sorted in. During the “Initial touch” phase, a window was pseudo-randomly illuminated for up to 30 s. Correct nose-poke responses to the illuminated window led to a brief acoustic click (2 kHz, 65 dB, 5 ms), the stimulus disappeared, and the magazine light was turned on. Upon head entry into the magazine, animals received two pellets for a correct response within 30 s or one pellet in the absence of a correct response after 30 s. Sessions lasted 30 min, and mice had to reach a criterion of 18 trials per session in two consecutive sessions to proceed to pre-training stages 3, 4, and 5. During stage 3 (“Must Touch”), mice had to respond to the stimulus window to receive a pellet, and an inter-trial interval (ITI) of 20 s was introduced after pellet delivery. During stage 4 (“Must initiate”), trials had to be initiated after the ITI through a head entry into the illuminated food magazine. In the final pre-training stage (“Punish incorrect”), mice learned to avoid the windows that were not illuminated, as a wrong response resulted in a 10-s timeout signaled by the house light. The timeout was followed by the ITI prior to the start of a correction trial (CT). During a CT, the stimulus was shown in the same window. CTs were repeated until a correct choice was made. In the pre-training stage 5, mice had to reach an accuracy of ≥80% in two consecutive sessions. Performance was defined as the number of correct responses divided by the total number of correct and incorrect trials and did not include CTs (Table 2).

**Table 2:** Touchscreen pre-training stages.

Stage	Session duration	Max. trials/session	Criteria	Description
Sorter habituation	—	—	2 hours and sorter visits per mouse ≥ 3	Sorter inactive 1 pellet rewarded for each food magazine head entry
Initial touch	30 min	18	reached max. trials in 2 consecutive sessions	Sorter active 2 pellets for a correct response 1 pellet in the absence of a correct response after 30-seconds stimulus duration
Must touch	15 min	18	¯ ″ ¯	Sorter active 1 pellet for a correct response
Must initiate	15 min	18	¯ ″ ¯	¯ ″ ¯
Punish incorrect	15 min	18	≥ 80% correct responses in 2 consecutive sessions	¯ ″ ¯

ˉ ″ ˉ: same as in the previous stage.

Throughout pre-training and all behavioral tasks, a mouse was allowed to complete up to seven sessions per day, but each session was followed by an intersession interval (ISI) of at least 90 min during which the sorter prevented the mouse from re-entering the operant chamber, thus providing time for other mice to enter the chamber. The end of a session was always signaled by an on–off flickering of the full screen (flicker count: 10, duration: 100 ms on/100 ms off, pause: 1 s), which was designed to motivate mice to leave the chamber.

### 2.4 TUNL task

**Figure 2:**
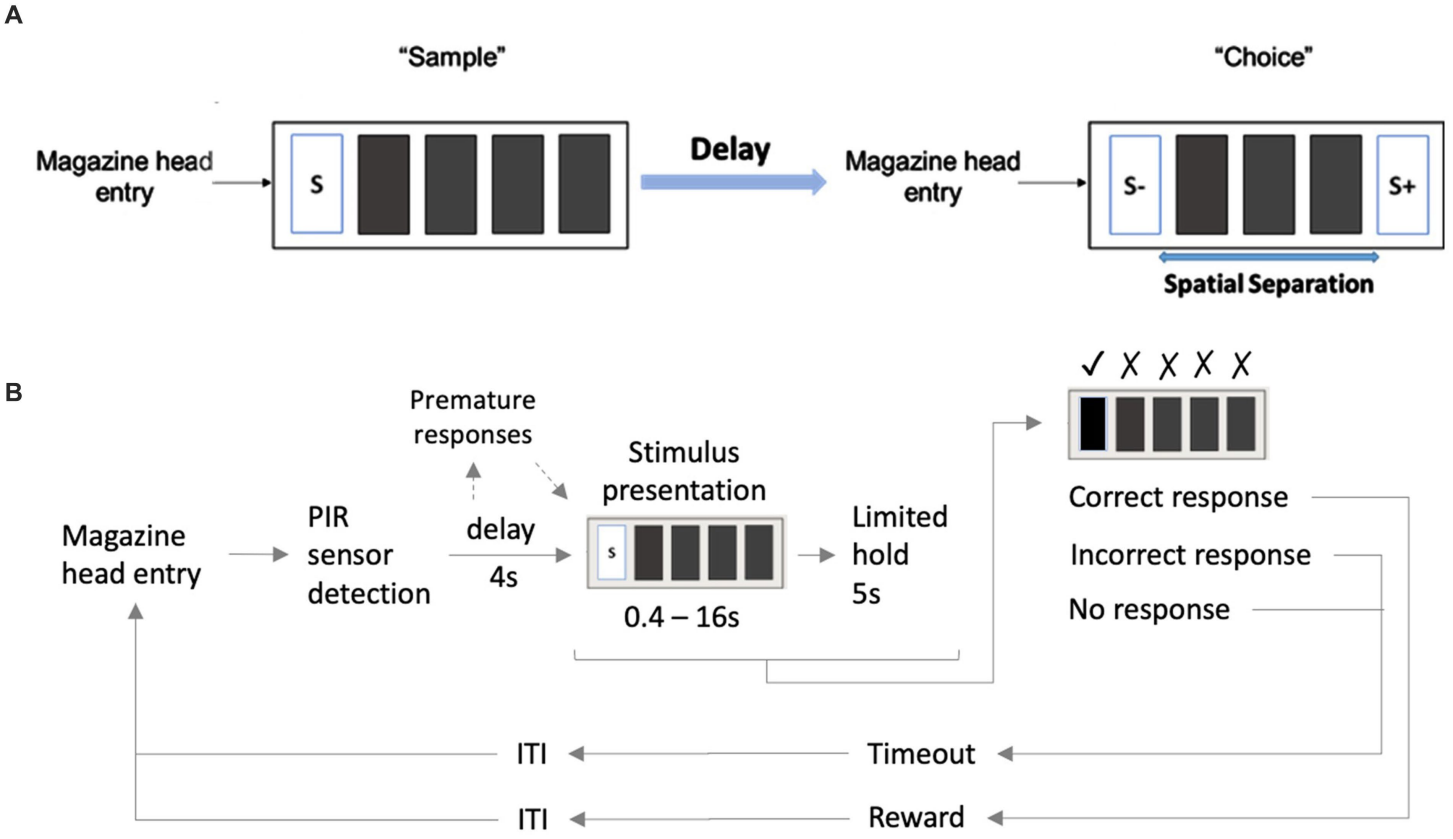
Structure of a TUNL task and 5-CSRTT trials. **(A)** TUNL task: rectangles represent five touchscreen response windows. Trials were initiated through a head entry into the food magazine. During the sample phase, mice had to respond to the lit window, which subsequently disappeared. During the choice phase only, a response to S+ was rewarded. The locations of S+ and S− were pseudo-randomly allocated for each trial. White rectangular: window lit (stimulus), dark rectangular: window dark (blank), S+: rewarded stimulus, S−: unrewarded stimulus. **(B)** 5-CSRTT task: a trial started when a mouse was detected at the divider after leaving the food magazine. Following a 4-s delay, a stimulus was presented in one of the five windows. Premature responses during the 4-s delay were recorded but did not result in abortion of the trial. A mouse was rewarded if it poked the window in which the stimulus appeared during stimulus presentation or within a 5-s limited hold period that started after the stimulus disappeared (correct choice). A response to a blank window (incorrect response) or no response (omitted trial) was followed by a 10-s timeout signaled by the house light. Reward collection or timeout was followed by a 20-s ITI before a new trial could be initiated. S: stimulus, ITI: inter-trial interval, PIR: passive infrared.

Performance in the TUNL task (Figure 2A) depends on spatial working memory and spatial pattern separation ability (Oomen et al., 2013). Trials were initiated through head entries into the illuminated food magazine. During the subsequent sample phase, mice had to respond to the S+ (illuminated window), which subsequently disappeared. Next, a delay period was included; after the delay, the magazine light went on again, and a second head entry initiated the choice phase. During the choice phase, two stimuli were presented, one at the previous location (now S−) and one at a new location (S+, Figure 2A). A nose-poke to either stimulus resulted in their disappearance, but only a correct response to the S+ was rewarded with a pellet. An incorrect choice (S−) resulted in a 10-s timeout (signaled by a click; 4 kHz, 65 dB, 5 ms, and the house light). Responses to non-illuminated windows were recorded but were without consequence. Reward collection or timeout was followed by an ITI (15 s), and sessions ended after 18 trials (excluding correction trials) or 15 min, whichever occurred first. The task consists of several training and test stages (Table 3), which vary in the number of response windows, the spatial separation labeled S0–S3 according to the number of windows between the rewarded stimulus (S+) and the unrewarded stimulus (S−) and the delay period between the sample and the choice phase.

**Table 3:** TUNL task training and test stages.

Stage		Stimuli location	Delay [s]	Criterion
TRAINING	Three windows	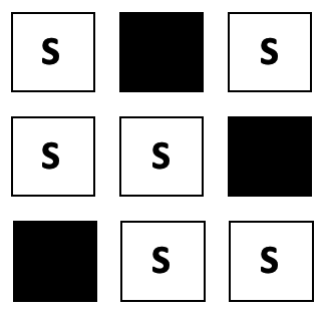	2	≥ 70% correct responses in 2 consecutive sessions
	S3	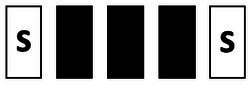	ˉ ″ ˉ	≥ 70% correct responses in 2 consecutive sessions
	S2	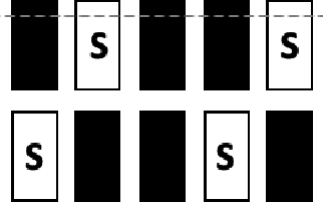	ˉ ″ ˉ	ˉ ″ ˉ
	S1	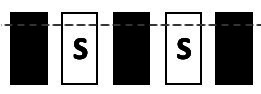	ˉ ″ ˉ	ˉ ″ ˉ
TEST	Mixed S1, S0; incl. central window	S1: 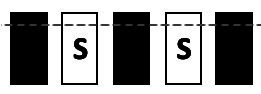 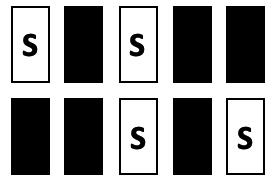 S0: all possible combinations incl.: 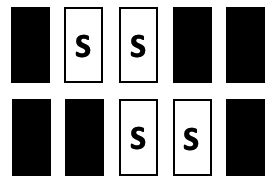	0	stable performance
	Test spatial separation		ˉ ″ ˉ	90 trials
	Test delay		0,3,6	140 trials

ˉ ″ ˉ: as in the previous stage.

To facilitate the acquisition, mice were first trained on a three-window mask, and head entries between sample and choice phases were rewarded randomly in 10% of the trials. At each stage, S+ and S− occurred pseudo-randomly in one of the indicated location options (Table 3). Mice moved individually from one stage to the next after reaching the criterion. Once a mouse showed stable performance (≤10% variation in individual performance over three consecutive sessions) during the mixed S1 and S0 training stages, the individual performance level under these conditions was measured over 90 trials (about five sessions). As the major test of spatial working memory, mice were subsequently tested over 140 trials on three different delays (0, 3, and 6 s) between the sample and choice phases using mixed S1 and S0 separations (Table 3). Since mice had to initiate the choice phase by head entry to the magazine after the delay period (Figure 2A), the actual time interval between poking the sample stimulus and the later appearance of the test stimuli could be longer.

The TUNL task was assessed by analyzing the following parameters: trials to criterion, performance defined as the percentage of correct responses (the number of correct responses divided by the total numbers of correct and incorrect trials; not including CTs), and perseveration score (the average number of CTs per incorrect response).

### 2.5 5-CSRTT

Animals were given a 1-week break between ending TUNL and beginning 5-CSRTT. The divider with a central opening was added to the operant chamber for the 5-CSRTT (Figure 1), and animals were re-trained on the “Punish incorrect” stage until they reached the criterion (Table 2), as previously described (Oomen et al., 2013). The 5-CSRTT followed the previously established task protocol (Robbins, 2002; Romberg et al., 2011; Mar et al., 2013; Remmelink et al., 2017) with some modifications, as shown in Figure 2B; Table 4. Trial initiation differed between cohorts: While cohort Y1 initiated a trial by nose-poking the illuminated food magazine, all subsequent cohorts had to be detected via the central opening of the divider to ensure an animal was facing the touchscreen at trial initiation. Following a 4-s delay period, the S+ (lid window) was shown at a random window location for a limited time period. Mice were rewarded if they responded to the S+ (correct response) either during stimulus presentation or within a 5-s limited hold period that began after the stimulus disappeared. Premature responses that occurred during the delay period and thus prior to stimulus presentation were recorded but not punished. A response to a blank window (incorrect response) or no response (omitted trial) led to a click (4 kHz, 65 dB, 5 ms), the house light turning on, and a 10-s timeout. After reward collection or timeout, there was a 20-s ITI before a new trial could be initiated by nose-poking the illuminated food magazine (cohort Y1) and crossing the divider (cohorts Y2, L 4-mo, and O1).

**Table 4:** 5-CSRTT task training and test stages.

Stages	Stimulus duration [s]	Session duration	Criteria
Training	16	15 min or 20 trials	≥80% accuracy, <30% omission in 2 consecutive sessions
*ˉ ″ ˉ*	8	*ˉ ″ ˉ*	*ˉ ″ ˉ*
*ˉ ″ ˉ*	4	*ˉ ″ ˉ*	*ˉ ″ ˉ*
*ˉ ″ ˉ*	2	*ˉ ″ ˉ*	*ˉ ″ ˉ*
Testing	Mixed SD	30 min or 25 trials	28 sessions

ˉ ″ ˉ: as in the previous stage, SD: stimulus duration. Mixed SDs: 4, 2, 1.6, 1, 0.8, 0.6, and 0.4 s.

Mice were trained on different stimulus durations in the following order: 16, 8, 4, and 2 s. For each stimulus duration, the training criteria were ≥ 70% correct responses and < 30% omissions in two consecutive sessions. Sessions lasted 15 min, or a maximum of 20 trials, whichever occurred first. Subsequently, mice were tested for 28 sessions on a set of mixed stimulus durations (4, 2, 1.6, 1, 0.8, 0.6, and 0.4 s), all of which occurred pseudo-randomly within one session. During this test phase, sessions lasted 30 min or 25 trials, whichever occurred first, resulting in approximately 100 trials for each stimulus duration.

Parameters analyzed to assess performance in the 5-CSRTT were trials to criterion, performance expressed as the percentage of correct responses (the number of correct responses divided by the total number of correct and incorrect trials), omissions (the number of omitted trials divided by the total number of trials), premature responses during the delay period (the total number of responses performed during the delay period), latencies to collect a reward (the average time needed to collect reward), and latencies of correct choices (the average time between stimulus onset and a correct nose-poke response).

### 2.6 Visual discrimination and reversal learning task

Mice were first trained to discriminate a pair of static shapes, a marble, and a fan that simultaneously occurred on the touchscreen upon trial initiation by a nose-poke into the illuminated food magazine (Mar et al., 2013). Reward contingencies were assigned pseudo-randomly, and a correct response to the S+ was rewarded with a pellet, while an incorrect nose-poke resulted in a 5-s timeout, signaled by a click (4 kHz, 65 dB, 5 ms) and the house light on. Incorrect responses were followed by correction trials until a correct choice was made. The ITI was 20 s, and sessions ended after 20 trials or 20 min, whichever occurred first.

After a mouse had reached the criterion consisting of ≥80% correct responses (not including CTs) in two consecutive sessions, it was moved to the second phase, during which the reward contingencies were reversed (Table 5) until the criterion was reached again.

**Table 5:** Visual discrimination and reversal learning task.

Stages	Session duration	ITI (s)	Timeout (s)	Criterion
VD	20 min or 20 trials	20	5	≥80% correct responses in 2 consecutive sessions
Reversal	*ˉ ″ ˉ*	*ˉ ″ ˉ*	*ˉ ″ ˉ*	*ˉ ″ ˉ*

ˉ ″ ˉ: as the previous stage, VD: visual discrimination, ITI: inter-trial interval.

The parameter analyzed to assess the performance was trials to criterion.

### 2.7 Data analysis

Data analysis and visualization were performed using GraphPad Prism (6.01) and R (R Core Team 2020). Animals were excluded from the analysis if they failed to reach the criterion at training or test stages or after they developed age-related reduced visual ability (see Supplementary material 7). A one-way ANOVA was performed to compare the effect of age on trials to criterion in the TUNL-3 W task. Group differences in latencies to correct responses and reward collection during 5-CSRTT acquisition were analyzed using the Wilcoxon rank-sum exact test. Group differences in the effect of center opening implementation during 5-CSRTT and the number of sessions to reach the criterion during TUNL S3 were analyzed using the Mann–Whitney *U* test. Generalized linear mixed models or generalized linear regressions were used to analyze TUNL and 5-CSRTT performance. Model predictions of estimated marginal means on the original response scale, as well as the model coefficient estimates and 95% CI of the main effects, are presented. For more detailed information on the models, please refer to Supplementary material 2.

## 3 Results

### 3.1 Using the fully automated touchscreen system with a sorter

From the habituation phase onwards, all mice readily passed through the sorter to the touchscreen compartment, resulting on average in 4.6 ± 0.26 individual sessions or 82 ± 15.7 individual trials per day during task acquisition (Supplementary Figures S1A–D). However, observations in cohort Y1 revealed that not all touchscreen nose-pokes were registered. A functional change that re-positioned the infrared photodetectors in front of the mask (Figure 1) resolved this issue and approximately halved the median number of sessions required to reach criterion during TUNL S3 training in cohorts Y2 and L 4-mo compared to cohort Y1 (Mann–Whitney *U* = 14.5, median = 5.5 and 19.5, respectively, *p* = 0.02; Supplementary Figure S2A). Furthermore, cohort Y1 exhibited high omission rates on the 5-CSRTT, which we believed to be caused by mice initiating trials before they were ready to pay attention. Forcing mice to face the touchscreen upon trial initiation reduced omission rates in cohorts Y2 and L 4-mo from approximately 40% to approximately 10% compared to cohort Y1 (Mann–Whitney *U* = 0.0, median = 0.11 and 0.43, respectively, *p*-values <0.0001; Supplementary Figure S2B).

### 3.2 Effect of age on TUNL acquisition

**Figure 3:**
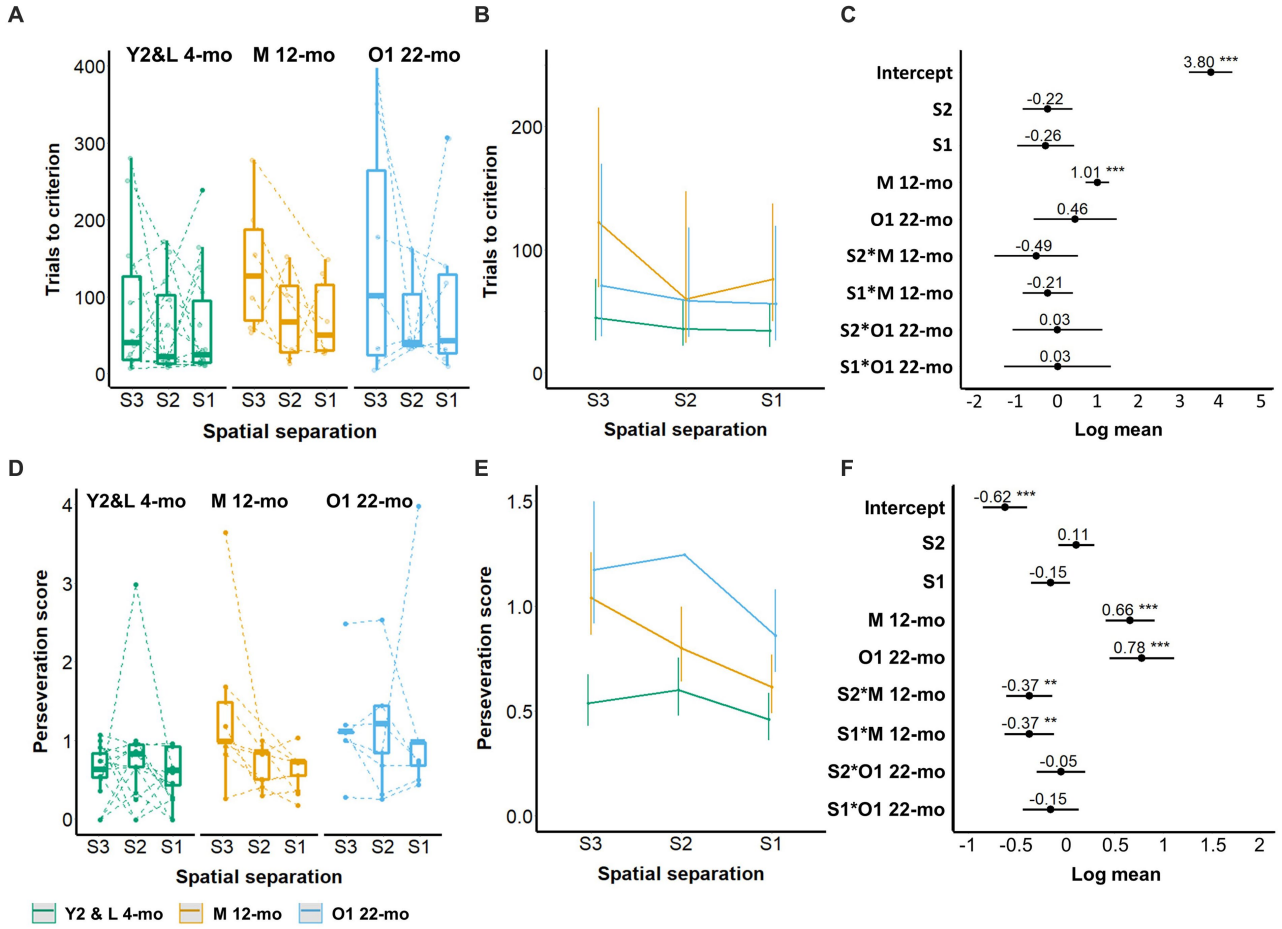
Effect of age on TUNL task acquisition. **(A–C)** Trials to criterion; **(D–F)** Perseveration score. **(A)** Trials to criterion for S3, S2, and S1 spatial separation. **(B)** Model prediction. Note the different scales of the *y*-axis in **A,B**. **(C)** Model coefficients of the age effect per spatial separation stage. **(D)** Perseveration score (average number of correction trials per incorrect response) for S3, S2, and S1 spatial separations. **(E)** Model prediction. Note the different scales of the *y*-axis in **D,E**. **(F)** Model coefficients of the aging effect per spatial separation stage. Y2 and L 4-mo *n* = 14; M 12-mo *n* = 9 (**A–C**, *n* = 6, 3 individuals were excluded (Supplementary material 6; Supplementary Figure S4) and 2 individuals were moved prematurely from S2 to S1); O1 22-mo *n* = 7. Symbols in **A,D** represent individual data. Error bars in **B,C** and **E,F** represent the 95% confidence interval (CI). Box plots in **A,D** show the 25th to 75th percentile, and whiskers extend to 1.5 × IQR. S: spatial separation.

Naive animals across cohorts Y2, L 4-mo, M 12-mo, and O1 22-mo required a median of 16 training days (minimum: 4 days, maximum: 37 days) from the start of three-window training to the completion of five-window training. No cohort differences were observed during the three-window training (Supplementary Figure S3). During five-window training, M 12-mo mice required more trials for S3 spatial separation acquisition than Y2 and L 4-mo mice (coefficient estimate (β) = 1.01, 95% confidence interval (CI) = 0.71 to 1.30, *Z*-value = 6.7, *p*-values <0.001; Figures 3A–C; Supplementary Table S1). Furthermore, M 12-mo and O1 22-mo mice repeated incorrect choices more often than the L 4-mo mice (β = 0.66 and β = 0.78, CI = 0.41 to 0.91 and 0.45 to 1.11, *Z*-value = 5.16 and 4.62, respectively, *p*-values <0.001; Figures 3D–F). The level of perseveration was independent of the spatial separation of stimuli for Y2 and L 4-mo and O1 22-mo mice (S2, S1 *p*s > 0.13; S2 × O1 22-mo, S1 × O1 22-mo *p*s > 0.2; Figure 3F; Supplementary Table S2). Only for M 12-mo mice, a significant interaction term indicated that perseveration decreased with shorter stimulus pair distances and thus training compared to the 4-month-old cohorts (S2 × M12-mo; β = −0.37, CI = −0.61 to −0.14, *Z*-value = −3.10, S1 × M12-mo; β = −0.37, CI = −0.63 to −0.12, *Z*-value = −2.89, *p*-values <0.01; Figure 3F; Supplementary Table S2).

### 3.3 Effect of age on TUNL probe trial

**Figure 4:**
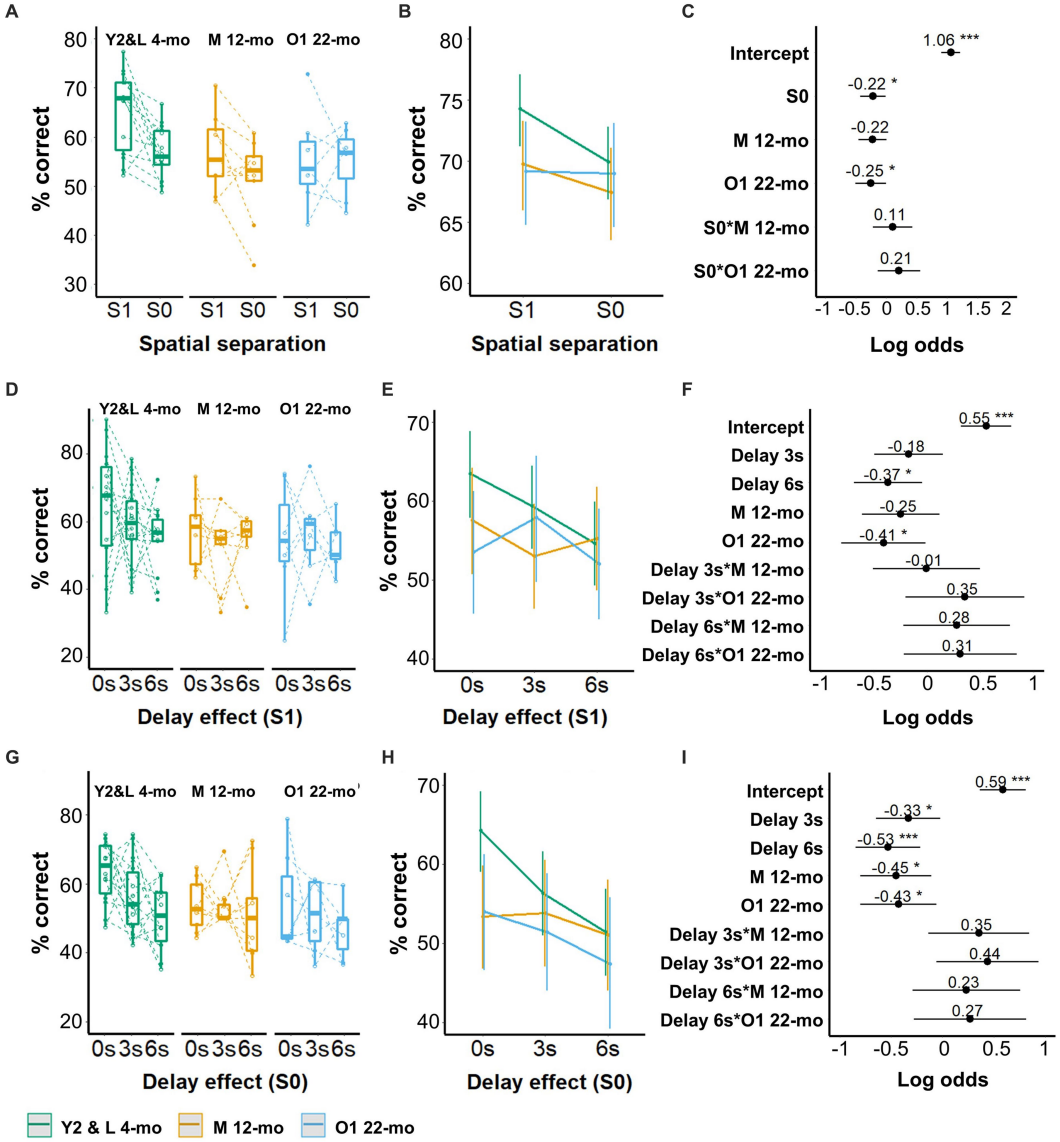
Effect of age on TUNL task performance. **(A)** Performance accuracy is depicted as the percentage of correct responses during mixed S1 and S0 spatial separation. **(B)** Model prediction. Note the different scales of the *y*-axis in **A,B**. **(C)** Model coefficients of the aging effect for mixed S1 and S0 spatial separation. **(D)** S1 performance (% correct responses) during delays. **(E)** Model prediction. Note the different scales of the *y*-axis in **D,E**. **(F)** Model coefficients of the aging effect for delays for S1. **(G)** S0 performance (% correct responses) during delays. **(H)** Model prediction. Note the different scales of the *y*-axis in **G,H**. **(I)** Model coefficients of the aging effect for delays for S0. Y2 and L 4-mo = 14; M 12-mo = 9; O1 22-mo (*n* = 7). Symbols in **A,D,G** represent individual data. Error bars in **B,C,E,F,H,I** represent a 95% confidence interval (CI). Box plots in **A,D,G** show the 25th to 75th percentile, and whiskers extend to 1.5 × IQR. S: spatial separation.

After task acquisition, the performance of mice on separation levels S1 and S0 was assessed in mixed sessions without imposed delay between the sample and choice phases, except for the intermittent magazine head entry. Y2 and L 4-mo mice performed with significantly higher accuracy on S1 compared to S0 trials (β = −0.22, CI = −0.43 to −0.01, *Z*-value = −2.01, *p* = 0.044), and this pattern was not significantly altered in any of the older cohorts (S0 × M12-mo, S0 × O1 22-mo *p*s > 0.2, Figure 4C; Supplementary Table S3). Although mice from both older cohorts appeared to perform with approximately 5% lower accuracy on S1 compared to L 4-mo mice, the model prediction was only significant for O1 22-mo (β = −0.25, CI = −0.50 to 0.00, *Z*-value = −1.96, *p* = 0.049; Figures 4A–C; Supplementary Table S3).

An introduction of delays to mixed S1 and S0 sessions revealed that, on S1 trials, 6 s but not 3 s delays had a significant effect on performance compared to no delay in Y2 and L 4-mo mice (β = −0.37, CI = −0.69 to −0.05, *Z*-value = −2.26, *p* = 0.041), and these slopes were not significantly altered in O1 22-mo and M 12-mo mice compared to Y2 and L 4-mo (*p*s > 0.2, Figures 4D–F, Supplementary Table S4). In line with the results obtained prior to delay introduction, only O1 22-mo mice had a lower performance than young mice at the S1 separation level (β = −0.41, CI = −0.81 to −0.02, Z-value = −2.05, *p* = 0.024; Figures 4D–F; Supplementary Table S4).

The effect of age increased as the distance between the stimulus pair was reduced to S0, making the task more difficult. At this separation level, Y2 and L 4-mo mice had lower accuracy levels after both 3- and 6-s time delays compared to no delay (β = −0.33, CI = −0.65 to −0.02, *Z*-value = −2.10, *p* = 0.036 and β = −0.53, CI = −0.85 to −0.22, *Z*-value = −3.31, *p* = 0.001, respectively), and the slopes of both older cohorts were not significantly different (*p*s > 0.08; Figures 4G–I; Supplementary Table S5). Furthermore, both M 12-mo and O1 22-mo mice had a lower performance than Y2 and L 4-mo mice at 0-s delay (β = −0.45, CI = −0.80 to −0.11, *Z*-value = −2.57, *p* = 0.010 and β = −0.43, CI = −0.80 to −0.06, *Z*-value = −2.26, *p* = 0.024, respectively; Figures 4G–I; Supplementary Table S5).

### 3.4 Effect of age on the 5-CSRTT acquisition

All mice trained on the 5-CSRTT had previously experienced the TUNL task.

**Figure 5:**
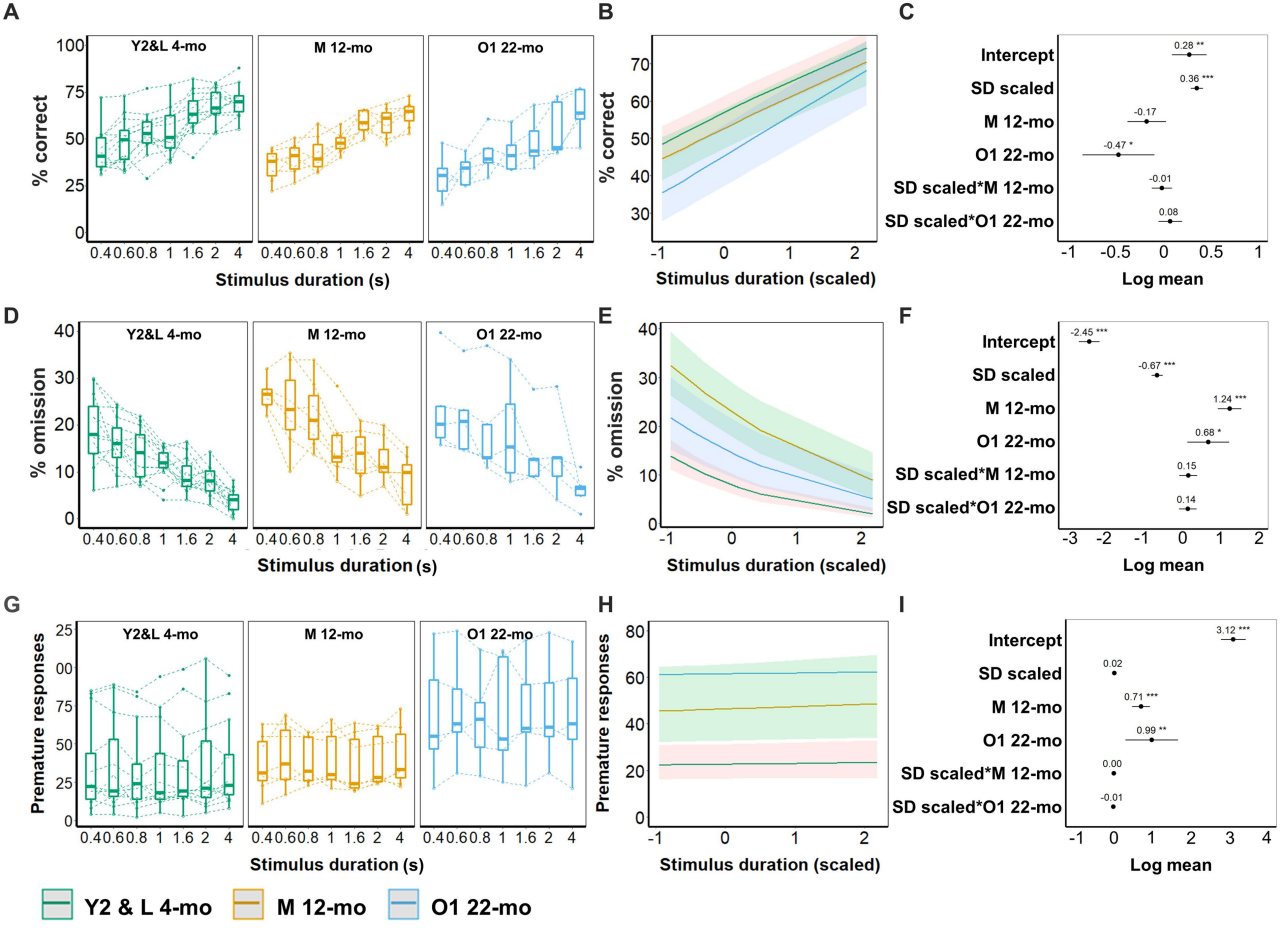
Effect of age on 5-CSRTT performance (SD; 0.4, 0.6, 0.8, 1, 1.6, 2, and 4 s). **(A)** Performance (% of correct responses) for the different durations. **(B)** Model predictions. Note the different scales of the *y*-axis in **A,B**. **(C)** Model coefficients estimate of the age effect on performance per stimulus duration. **(D)** Omission for the different durations. **(E)** Model predictions. Note the different scales of the *y*-axis in **D,E**. **(F)** Model coefficients estimate of the age effect on omission per stimulus duration. **(G)** Premature responses for the different durations. **(H)** Model predictions. Note the different scales of the *y*-axis in **G,H**. **(I)** Model coefficients estimate of the age effect on premature responses per stimulus duration. Y2 and L 4-mo = 14; M 12-mo = 7; O1 22-mo = 5. Symbols in **A,D,G** show individual data. Shaded areas in **B,E,H** and error bars in **C,F,G** represent a 95% confidence interval (CI). Box plots in **A,D,G** show the 25th to 75th percentile, and whiskers extend to 1.5 × IQR.

With shortened stimulus durations, young animals (Y2 and L 4-mo) required more trials to reach criterion (β = −0.7, CI = −1.19 to −0.21, *Z*-value = −2.81, *p* = 0.005; Figures 5A–C; Supplementary Table S6). Compared to young cohorts, both older cohorts (M 12-mo, O1 22-mo) required considerably more trials to reach the criteria (*p*-values <0.001). Furthermore, slopes significantly differed between the young and both older cohorts (SD_(scaled)_ × M 12-mo; β = 0.75, CI = 0.51 to 1.00, *Z*-value = 6.05, SD_(scaled)_ × O1 22-mo; β = 0.68, CI = 0.40 to 0.96, Z-value = 4.81; *p*-values <0.001), indicating that in older mice the number of trials to reach criterion seemed independent of stimulus duration (Figures 5A–C; Supplementary Table S6).

**Figure 6:**
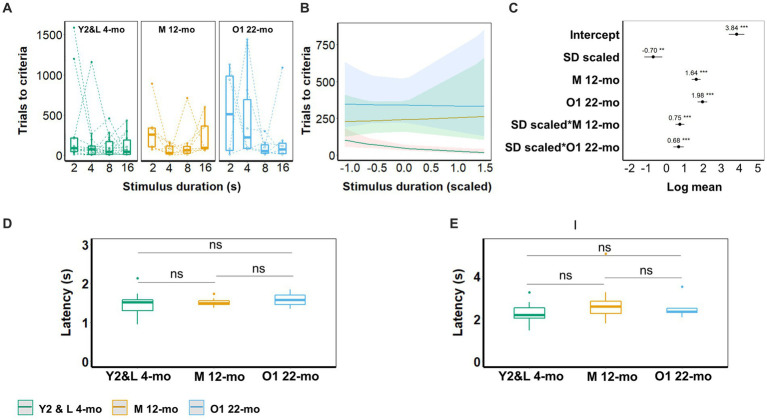
5-CSRTT acquisition. **(A)** Trials to criterion for the different stimulus durations (SD; 16, 8, 4, and 2 s). Symbols represent individuals. **(B)** Model predictions. Note the different scales of the *y*-axis in **A,B**. **(C)** Model coefficients estimate of the effect of age on the number of trials to criterion per stimulus duration. **(D)** Latency for correct choices during 2-s stimulus duration trials. **€** Latency to collect reward during 2 s stimulus duration trials. Box plots in **A,D,E** show the 25th to 75th percentile, and whiskers extend to 1.5 × IQR. Y2 and L 4-mo = 14; M 12-mo = 7; O1 22-mo: 6–7. One 22-mo subject had to be excluded from the 4-s training condition onward due to the development of visual impairments. Symbols in **A** represent individual data. Shaded areas in **B** and error bars in **C** represent the 95% confidence interval (CI).

The stimulus–response latencies and reward collection latencies did not differ between cohorts during correct trials at the final training stage (2 s) (Figures 5D,E).

### 3.5 Effect of age on 5-CSRTT performance during mixed stimulus duration sessions

Stimulus duration significantly affected both accuracy and omissions in Y2 and L 4-mo mice. Accuracy was higher (β = 0.36, CI = 0.29 to 0.42, *Z*-value = 10.87, *p*-values <0.001, Figures 6A–C; Supplementary Table S7), and omission rates were lower for longer stimulus durations (β = −0.67, CI = −0.81 to −0.52, *Z*-value = −8.95, *p*-values <0.001, Figures 6D–F; Supplementary Table S8), while premature responses were not affected (Figures 6G–I; Supplementary Table S9). The effect of stimulus duration on accuracy, omissions, and premature responses in M 12-mo and O1 22-mo mice did not differ from Y2 and L 4-mo mice (all *p*s > 0.19, Figure 6; Supplementary Tables S7–S9).

Age affected both accuracy and omissions. O1 22-mo, but not M 12-mo mice, performed with lower accuracy than Y2 and L 4-mo mice (β = −0.47, CI = −0.84 to −0.09, *Z*-value = −2.42, *p* = 0.015 and β = −0.17, CI = −0.37 to 0.03, *Z*-value = −1.64, *p* = 0.1, respectively, Figures 6A–D; Supplementary Table S7). Furthermore, omissions were higher in both 12- and 22-mo mice (β = 1.24, CI = 0.93 to 1.55, *Z*-value = 7.80, *p*-values <0.001 and β = 0.68, CI = 0.13 to 1.23, *Z*-value = 2.42, *p* = 0.016, respectively; Figures 6D–F; Supplementary Table S8). Age further increased premature responses in M 12- and O1 22-mo mice compared to Y2 and L 4-mo mice (β = 0.71, CI = 0.48 to 0.95, *Z*-value = 6.03, *p*-values <0.001 and β = 0.99, CI = 0.31 to 1.68, *Z*-value = 2.85, *p* = 0.004, respectively; Figures 6G–I; Supplementary Table S9).

### 3.6 Longitudinal assessment of TUNL task performance

**Figure 7:**
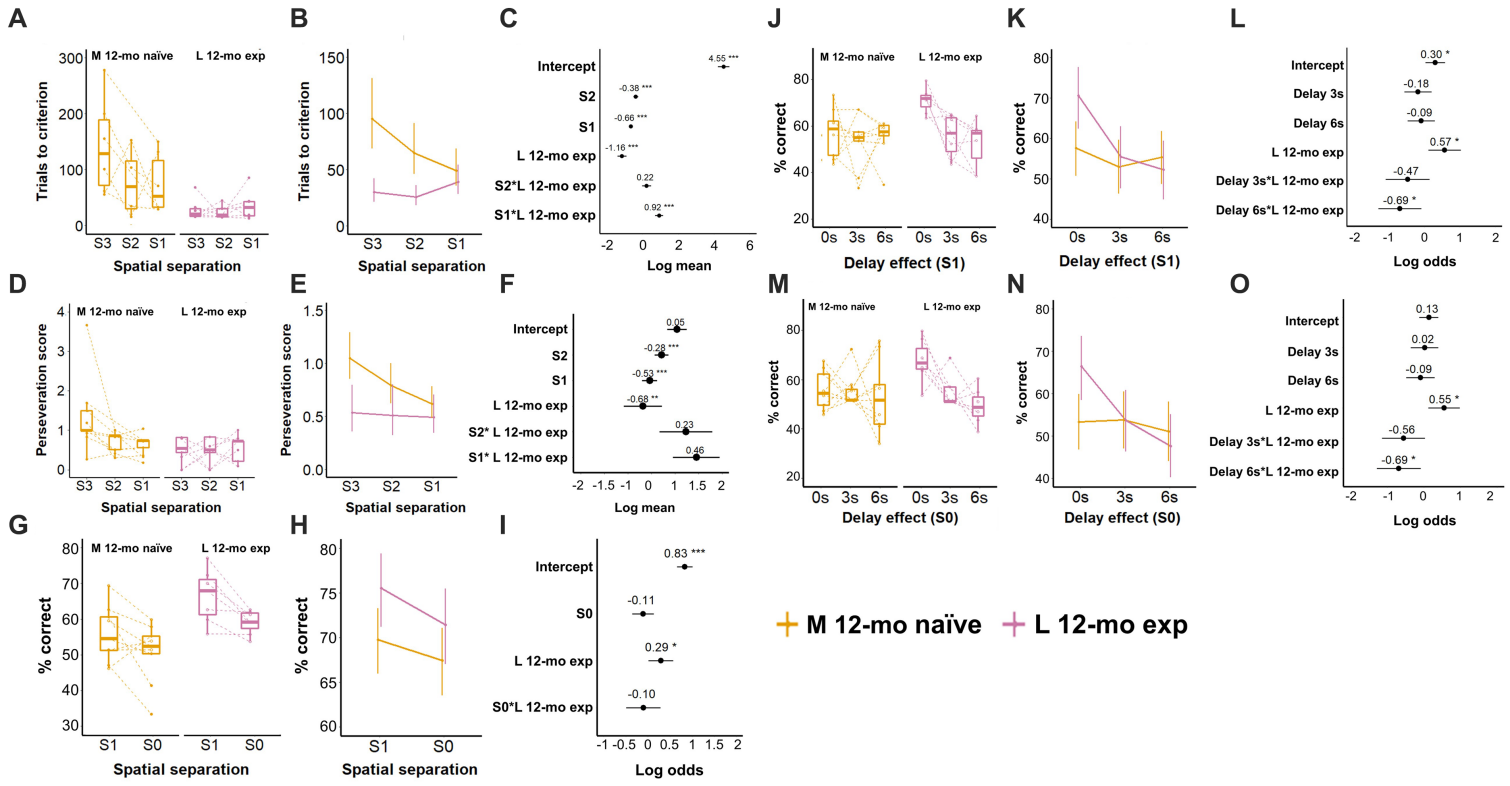
Effect of prior experience on TUNL task performance. **(A)** Trials to criterion for S3, S2, and S1 spatial separation. N per cohort: 12-mo experienced: 7, 12-mo naive: 6. **(B)** Model prediction. Note the different scales of the *y*-axis in **A,B**. **(C)** Model coefficients of the aging effect per spatial separation stage. **(D)** Perseveration score (average number of correction trials per incorrect response) for S3, S2, and S1 spatial separations. **€** Model prediction. Note the different scales of the *y*-axis in **D,E**. **(F)** Model coefficients. **(G)** Performance (% of correct responses) during mixed S1 and S0 spatial separation. **(H)** Model prediction. Note the different scales of the *y*-axis in **G,H**. **(I)** Model coefficients of the aging effect for mixed S1 and S0 spatial separation. **(J)** Performance (% of correct responses) during delays for S1. **(K)** Model prediction. Note the different scales of the *y*-axis in **J,K**. **(L)** Model coefficients of the aging effect for delays for S1. **(M)** Performance (% of correct responses) for S0 spatial separation. **(N)** Model prediction. Note the different scales of the *y*-axis in **M,N**. **(O)** Model coefficients of the aging effect for delays for S0. L 12-mo mice experienced *n* = 7; M 12-mo experienced *n* = 9 (**A–C**, *n* = 6), three individuals were excluded (Supplementary material 6; Supplementary Figure S4), and two individuals were moved prematurely from S2 to S1. Symbols in **A,D,G,J,M** show individual data. Error bars in **B,C,E,F,H,I,K,L,N,O** represent a 95% confidence interval (CI). S: spatial separation. Box plots in **A,D,G,J,M** show the 25th to 75th percentile, and whiskers extend to 1.5 × IQR.

Cohort L was re-tested on the TUNL task at 12-mo ≈ 34 weeks after the last TUNL exposure and ≈28 weeks after the last 5-CSRTT exposure. Comparison of L 12-mo experienced mice with naive mice of the same age (M cohort) revealed that, as expected, experienced mice needed fewer trials for task re-acquisition than naive mice required for acquisition on S3 spatial separation (β = −1.16, CI = −1.41 to −0.91, *Z*-value = −9.00, *p*-values <0.001; Figures 7A–C; Supplementary Table S10). However, M naive mice markedly reduced the number of trials to criterion as they progressed through training from S3 to S1 spatial separation (β = −0.66, CI = −0.77 to −0.54, *Z*-value = −11.24, *p*-values <0.001), while the same decline was not observed in L 12-mo mice (S1 × L 12-mo exp.; β = 0.92, CI = 0.70 to 1.15, *Z*-value = 8.11, *p*-values <0.001; Figures 7A–C; Supplementary Table S10).

During training, L 12-mo mice also repeated incorrect choices less frequently than M naive mice (β = −0.68, CI = −1.09 to −0.27, *Z*-value = −3.269, *p* = 0.001), a difference that also seemed to disappear when M naive and L 12-mo exp. reached S1 separation training (Figures 7D–F; Supplementary Table S11).

After both cohorts had been trained to criterion, experienced L 12-mo mice performed spatial separation testing (mixed session S1 and S0, 0-s delay) with higher accuracy than M naive mice on S1 trials (β = 0.29, CI = 0.01 to 0.58, *Z*-value = 2.03, *p* = 0.043; Figures 7G–I; Supplementary Table S12). No further significant differences were observed. The higher accuracy of L 12-mo mice on S1 and S0 trials without delay was maintained after the addition of delay trials (β = 0.57, CI = 1.1 to 1.04, Z-value = 2.42, *p* = 0.015 and β = 0.55, CI = 0.12 to 0.98, *Z*-value = 2.5, *p* = 0.012, respectively; Figures 7J–O; Supplementary Tables S13, S14). However, at 6-s delay trials, accuracy levels declined on both S1 and S0 trials in L 12-mo mice compared to M naive mice (β = −0.69, CI = − 1.31 to −0.09, *Z*-value = −2.24 and β = −0.69, CI = −1.29 to −0.09, *Z*-value = −2.25, respectively; *p*s < 0.03), whose accuracy levels remained relatively stable (*p*s > 0.6; Figures 7J–L; Supplementary Tables S13, S14), as a result, performance levels for both cohorts were around chance level at 6-s delay trials (S1 ∼ 55%, S0 ∼ 50% accuracy).

A comparison of the performance of the L cohort aged 12 months with their own performance at 4 months revealed differences only during the TUNL (re-) acquisition stage but not during the spatial separation and delay testing, indicating preservation of cognitive abilities (data not shown).

### 3.7 Longitudinal assessment of 5-CSRTT task performance

**Figure 8:**
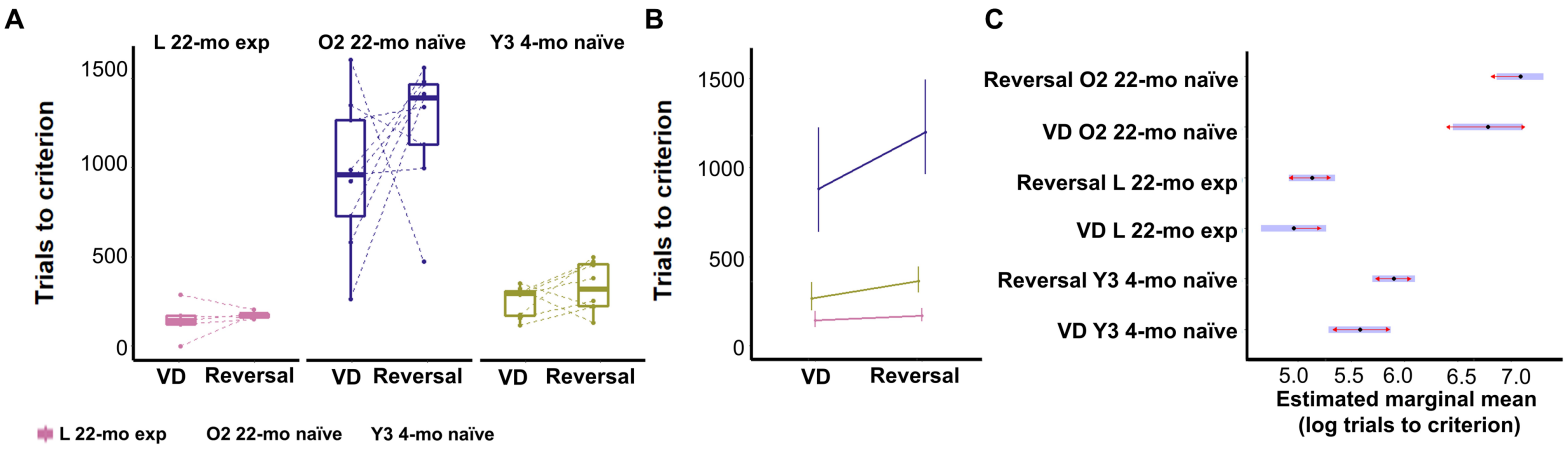
Effect of age and prior touchscreen experience on visual discrimination and reversal learning. **(A)** Trials to criterion per phase, first visual discrimination and then its reversal. Symbols show individual data. **(B)** Model prediction. Error bars represent 95% CI. **(C)** Estimated marginal mean and 95% CI of prior experience on associative and reversal learning. Blue bars represent confidence intervals. Red lines allow marginal mean comparisons; if arrows between cohorts overlap, the difference is not significant. Y3 4-mo = 8, L 22-mo experienced *n* = 5, O2 22-mo naive *n* = 9.

5-CSRTT task re-acquisition in L 12-mo mice required fewer trials than task acquisition in M naive mice (β = −1.61, CI = −1.83 to −1.38, *Z*-value = −14.03, *p*-values <0.001; Figures 8A–C; Supplementary Table S15). Moreover, the stimulus duration-dependent effects differed significantly between both cohorts (β = −0.75, CI = −0.99 to −0.51, *Z*-value = −6.13, *p*-values <0.001; Figures 8A–C; Supplementary Table S15), as L 12-mo mice had a tendency to require fewer trials with longer stimulus durations compared to M naive mice that had a tendency to require more trials consistent with it being the beginning of training.

**Figure 9:**
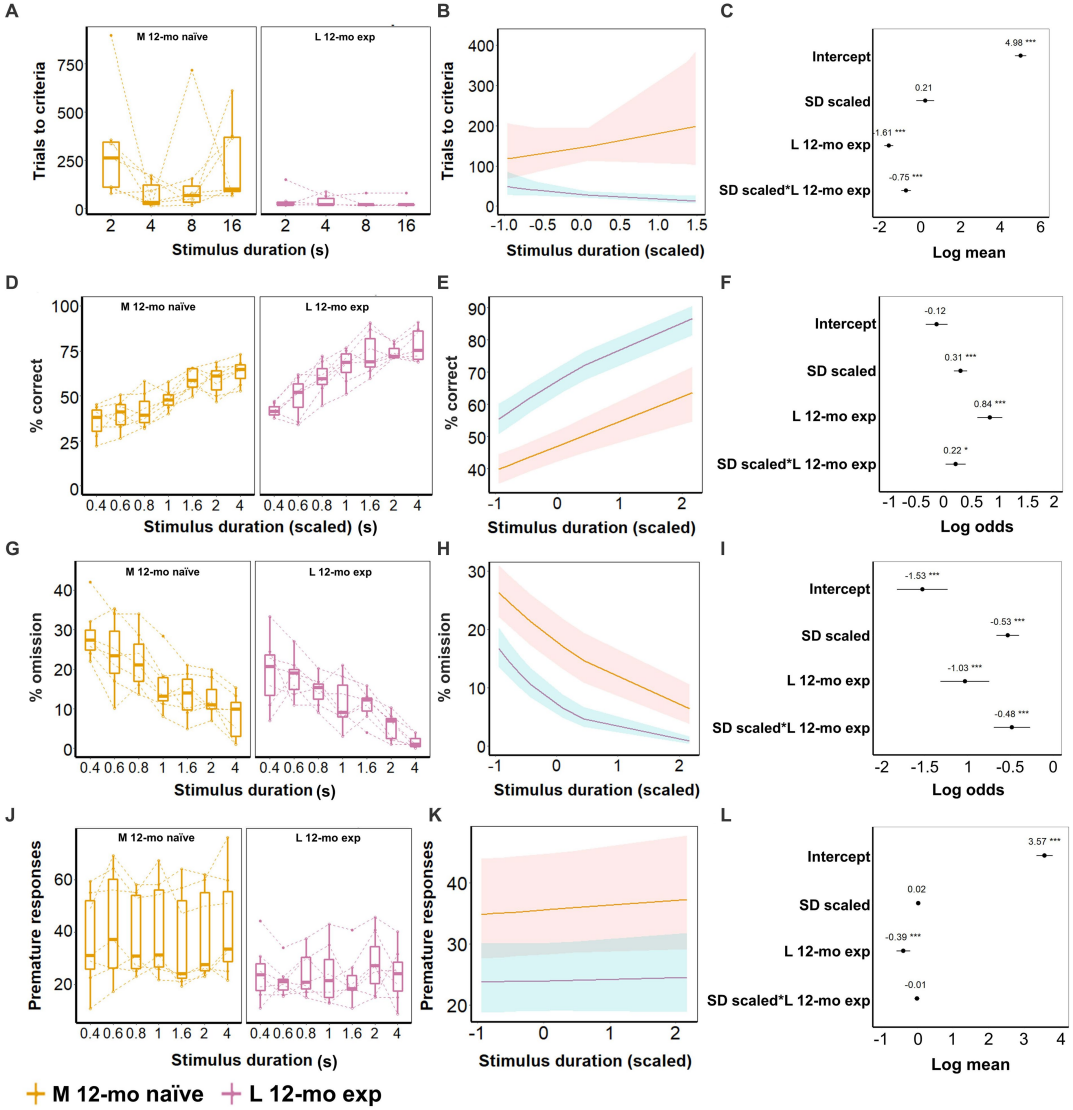
Effect of prior experience on 5-CSRTT task performance. **(A–C)** Task acquisition (SD; 16, 8, 4, and 2 s); **(D–L)** Performance on 5-CSRTT task (SD; 4, 2, 1.6, 1, 0.8, 0.6, and 0.4 s). **(A)** Trials to criterion for the different durations. **(B)** Model predictions. Note the different scales of the *y*-axis in **A,B**. **(C)** Model coefficients estimate of the age effect on the number of trials to criterion per stimulus duration. **(D)** Performance (% of correct responses) for the different durations. **(E)** Model predictions. Note the different scales of the *y*-axis in **D,E**. **(F)** Model coefficients estimate and 95% CI of the age effect on performance per stimulus duration. **(G)** Omission for the different durations. **(H)** Model predictions. Note the different scales of the *y*-axis in **G,H**. **(I)** Model coefficients estimate of the age effect on omission per stimulus duration. **(J)** Premature responses for the different durations. **(K)** Model predictions. Note the different scales of the *y*-axis in **J,K**. **(L)** Model coefficients estimate of the age effect on premature responses per stimulus duration. Data of seven mice/cohort. Symbols in **A, D, G**, and **J** show individual data. Shaded areas in **B,E,H,K** and error bars in **C,F,G,L** represent the 95% confidence interval (CI). Box plots in **A,D,G,J** show the 25th to 75th percentile, and whiskers extend to 1.5 × IQR.

After all animals had been trained to criterion, L 12-mo mice still performed better overall during the subsequent test phase compared to M-naive mice. Experienced mice had higher accuracy (β = 0.84, CI = 0.61 to 1.06, *Z*-value = 7.31, *p*-values <0.001; Figures 8D–F), lower omission (β = −1.03, CI = −1.31 to 0.75, *p*-values <0.001; Figures 8G–I), and fewer premature responses (β = −0.39, CI = −0.59 to 0.20, *p*-values <0.001; Figures 8J–L) compared to M naive mice (Supplementary Tables S16–S18). Furthermore, at this test stage, M naive mice showed increased accuracy and decreased omission levels with longer stimulus durations (*p*-values <0.001) although this effect was still slightly more pronounced in L 12-mo mice compared to M naive mice (β = 0.22, CI = 0.04 to 0.40, *Z*-value = 2.4, *p* = 0.016 and β = −0.48, CI = −0.69 to −0.27, *p*-values <0.001, respectively; Figures 8D–I).

5-CSRTT task re-acquisition for L 12-mo mice was faster and required fewer trials (β = −0.87, CI = −0.95 to −0.79, *Z*-value = −20.77, *p*-values <0.001; Supplementary Table S19), especially during long stimulus durations (SD_(scaled)_ × L12-mo exp.; β = −0.42, CI = −0.51 to −0.33, *Z*-value = −9.363, *p*-values <0.001) compared to the initial acquisition at 4 months. During the test phase, L 12-mo mice had a higher performance than L 4-mo (β = 0.43, CI = 0.33 to 0.52, *Z*-value = 8.70, *p*-values <0.001; Supplementary Table S20), and at longer stimulus durations, it made fewer omissions than L 4-mo (β = −0.44, CI = −0.65 to −0.24, *Z*-value = −4.22, *p*-values <0.001; Supplementary Table S21). However, L 12-mo mice showed more premature responses than L 4-mo mice (β = 0.26, CI = 0.17 to 0.36, *Z*-value = 5.47, *p*-values <0.001; Supplementary Table S22).

### 3.8 Effect of age and prior touchscreen task experience on visual discrimination and reversal learning task at 22 months

Touchscreen task-experienced L 22-mo mice with no prior experience of the visual discrimination task required less trials to reach criterion than both O2 22-mo naive and Y3 4-mo naive mice (estimated marginal mean contrast (emmc) = −1.82 and − 0.62, CI = −1.28 to −2.37 and − 0.84 to −0.40, Z-value = 7.98 and 6.84, respectively; *p*-values <0.0001; Figures 9A–C; Supplementary Table S23).

L 22-mo also reversed faster than both O2 22-mo (emic = −1.96, CI = −1.58 to −2.33, *Z*-value = 12.43, *p*-values <0.0001) and even Y3 4-mo naive mice (emic = −0.77, CI = −0.95 to −0.58, *Z*-value = 9.92, *p*-values <0.0001; Figures 9A–C; Supplementary Table S23).

## 4 Discussion

This study evaluated the cognitive abilities of mice, focusing on age-related variations. It revealed how cognitive training in longitudinal behavioral studies influenced performance outcomes during subsequent testing. Additionally, the study highlighted the beneficial effects of repetitive training on cognition in older subjects. The research was significantly enhanced by our home-cage-based testing system, where group-housed mice, without any food restrictions, voluntarily engaged in challenging touchscreen cognitive tasks. Consequently, we established a minimally invasive testing environment, which we believe holds substantial promise for high-throughput behavioral studies.

### 4.1 Automated home-cage-based touchscreen 5-CSRTT and TUNL tasks

Home-cage-based testing depends on the animal’s voluntary participation. Our data indicate that mice consistently returned to the sorter and operant chamber to engage in behavioral tasks. In cohort Y1, we noted that the nose-pokes at the peripheral windows were initially too shallow for detection. However, repositioning the infrared (IR) grid in front of the mask significantly improved nose-poke detection and reduced the number of sessions needed to reach the criterion. Mice from various age cohorts completed the TUNL task training in an average of 19 days, a duration comparable to the 13–21 days reported for young mice around 3 months of age (Supplementary Table S24) (Kim et al., 2015; Houlton et al., 2021; Dexter et al., 2022). We also successfully addressed the commonly high omission rates in the mouse 5-CSRTT (Fletcher et al., 2007; Sanchez-Roige et al., 2012; Young et al., 2013), often reported as 20% or higher. By ensuring mice were detected at both the food magazine and the newly added central chamber divider, they faced the touchscreen at the start of each trial, which helped reduce omission rates to approximately 10%. The reduction was achieved without extended periods of food deprivation, and we believe this modification could also enhance the efficiency of the 5-CSRTT in conventional setups.

Fewer omissions likely contributed to the rapid acquisition of the 5-CSRTT observed: L 4-mo mice completed 5-CSRTT training in an average of 12 days, significantly faster than the conventional training period, which can exceed 50 days (Supplementary Table S25; Humby et al., 2005; Romberg et al., 2011; McTighe et al., 2013; Remmelink et al., 2017). Interestingly, in a similar home-cage-based study with single-housed animals, mice completed all training stages of the 5-CSRTT within just 4 days (Remmelink et al., 2017). However, a direct comparison between these different setups is difficult as training and testing protocols differed, for example, in terms of session lengths, criteria, and prior experience of the animals (Supplementary Tables S24, S25).

It is worth noting that while avoiding food deprivation and labor-intensive handling, the design of sorter-based home-cage studies requires careful consideration of factors such as session length, inter-session interval, and maximum number of sessions per day. The consideration is essential to sustain motivation and afford opportunities for all mice to participate in the task. However, our system offers the advantage of supporting group housing, which avoids the need for single-house social animals and facilitates the use of one operant chamber for multiple animals in high-throughput studies.

### 4.2 Effect of age on spatial learning and attention in the home-cage-based touchscreen system

The detrimental effect of aging on rodent spatial working memory is well documented in both conventional and touchscreen-based memory tests (Pepeu, 2004; Fahlström et al., 2011; Weber et al., 2015; Shoji et al., 2016; Yanai and Endo, 2021; Smith et al., 2022). Our home-cage-based touchscreen system successfully identified age-related working memory deficits. During TUNL acquisition, naive 12- and 22-month-old mice showed a higher tendency for incorrect choices (perseverance) compared to 4-month-old mice. In the test phase, 22-month-old mice exhibited lower accuracy in mixed S0 and S1 sessions without delays compared to 4-month-old mice, while both 12- and 22-month-old mice showed reduced accuracy compared to 4-month-old mice when the difficulty was further increased by the addition of delay trials. These findings align with previous studies reporting increased perseverance, compulsivity, and decreased performance in spatial working memory tasks in aged rodents and humans (Pepeu, 2004; Fahlström et al., 2011; Slachevsky et al., 2011; Weber et al., 2015; Shoji et al., 2016; Yanai and Endo, 2021; Smith et al., 2022).

Interestingly, age-related differences in performance seemed to diminish with longer delays, despite the increased difficulty. This trend is likely a flooring effect, as performance levels on 0-s delay trials decreased by 5–15% when delay trials were added, particularly in the two older cohorts, and continued to decline with increased delays, reaching accuracy levels of approximately 50%. Similarly, performance in 6-s delay trials has been noted in past studies, with some mice performing at chance level (Kim et al., 2015; Shepherd et al., 2021). To mitigate such flooring effects, future studies may consider reducing difficulty by including only one delay and testing it at a single separation level per session. Nevertheless, the observed age-dependent decline in performance underscores the system’s sensitivity to age-related deficits in working memory.

In addition to working memory, age may also impact sustained attention and impulsivity in both rodents and humans (West, 1996; Mani et al., 2005; Isella et al., 2008; Bordner et al., 2011; Harati et al., 2011; Zanto and Gazzaley, 2014; Sakurai et al., 2020). Here, 12- and 22-month-old naive mice needed more trials to reach the criterion during 5-CSRTT training than 4-month-old naive mice. Despite the task’s time-critical nature, this effect was likely not due to altered locomotor abilities, as the latencies to correct responses and reward collection did not differ between cohorts. During the test phase, 22-month-old mice had lower accuracy levels, and both 12- and 22-month-old mice exhibited higher omission rates and more premature responses compared to 4-month-old mice. Similar trends were observed in rats, where middle-aged and old-aged rats showed higher omission rates compared to young rats although accuracy was only decreased in old-aged rats (Harati et al., 2011). Few studies have examined middle-aged mice on the 5-CSRTT, possibly due to its labor-intensive nature in conventional setups. However, our results in middle-aged mice, in line with observations for rats, suggest that omission rates may be a sensitive measure for detecting early signs of age-related attentional deficits. The elevated premature responses in 12-month-old mice also imply that age-related impulsivity may precede attention deficits detected by accuracy levels in the 5-CSRTT. These findings are consistent with the notion that impulsivity is a risk factor preceding cognitive decline (Bateman et al., 2020). Although not all cohorts were tested simultaneously due to the study’s design, the observed age-dependent decline in impulsivity and attention corroborates previous findings. The home-cage-based methodology may help minimize cohort effects by eliminating stress from handling and increasing the variation in external test conditions within a cohort though this requires further systematic exploration. Nevertheless, these results affirm the test’s sensitivity to age-related cognitive decline within our home-cage-based setup.

### 4.3 Effect of prior experience on age-related cognitive decline

In longitudinal behavioral studies, it is important to consider the influence of training. Extensive training might obscure or mitigate early signs of cognitive decline in later life (Salthouse, 2009; Zarnani et al., 2020). In our study, we evaluated the performance of 12-month-old mice that had previously undergone both TUNL and 5-CSRTT training, comparing them to 12-month-old naive mice. As expected, the experienced mice reacquired the tasks more rapidly than the naive mice. Moreover, following successful training to criterion, the experienced 12-month-old mice continued to outperform their naive counterparts during no-delay trials on both S0 and S1 spatial separations. However, the introduction of delays led to a reduction in performance in both cohorts, potentially concealing differences between the cohorts. Additionally, in the 5-CSRTT, the experienced 12-month-old mice displayed higher accuracy and lower rates of omissions and premature responses, indicating better-sustained attention and reduced impulsivity compared to the naive mice of the same age. These findings suggest that despite a break in training and both cohorts being trained to criterion prior to testing, regular engagement in cognitive tasks had a discernible impact on performance in the longitudinal cohort.

### 4.4 Life-long training in touchscreen tasks may have a generalized positive effect on cognitive aging

The study also explored the cognitive performance of mice experienced in touchscreen tasks through a new challenge—the visual discrimination learning and reversal task. This task, focusing on behavioral flexibility, had not been previously tackled in earlier tasks. The comparison of naive 4- and 22-month-old mice highlighted the detrimental effects of aging, as older mice needed more trials for both acquiring and reversing the task. These findings align with existing research in humans and rodents on the impact of age on associative and reversal learning (Brushfield et al., 2008; Weiler et al., 2008; Patel and Larson, 2009; Buscher et al., 2017). Prior studies with older mice, aged 17 to 24 months, showed diminished performance in reversal tasks during visual or olfactory discrimination (Patel and Larson, 2009; Buscher et al., 2017), mirroring human studies where elderly participants struggled with reward-based associative learning tasks, reversal learning, and transfer learning compared to their younger counterparts (Weiler et al., 2008). Notably, however, 22-month-old mice with prior experience significantly outperformed both their naive age-mates and even younger 4-month-old naive mice. This finding parallels human studies, where higher levels of education and lifestyle choices correlate with better individual cognitive abilities (Kujawski et al., 2018), leading to the concept of “super-agers” (Sun et al., 2016; Borelli et al., 2018), older individuals who display memory abilities surpassing those of younger people. The reasons for such exceptional performance are not entirely clear.

The familiarity with the touchscreen setup might have played a role, especially during the task’s initial acquisition phase. Additionally, although the TUNL and 5-CSRTT tasks did not directly target behavioral flexibility, the transition between these tasks could have inadvertently trained this ability. Noteworthy is the previous finding that experience with the TUNL task improved performance in the Morris Water Maze (Shepherd et al., 2021), indicating that touchscreen training can impact tasks in the same cognitive domain, like spatial working memory, even in drastically different testing environments. In that study, mice with TUNL experience also showed increased hippocampal neurogenesis, linked to enhanced reversal learning abilities (Anacker and Hen, 2017). Our results suggest that the benefits of life-long cognitive touchscreen training might extend beyond the targeted cognitive domains, warranting further research across various cognitive areas to uncover underlying mechanisms.

In conclusion, our study demonstrates that long-term touchscreen-based training not only maintains but also boosts cognitive performance in older mice. It highlights the nuanced ways in which touchscreen task training can influence subsequent behavior, possibly across cognitive domains, emphasizing the need for careful interpretation of results from longitudinal touchscreen studies. In addition, the study also underscores the potential of our setup and tasks for researching the effects of life-long cognitive training in mice.

Our low-stress, voluntary learning approach, combined with improvements to the experimental setup, contributed to the successful performance of both young and older mice. This method is promising for further exploration of other domains of age-related cognitive decline using different behavioral tasks adaptable to a fully automated system. Moreover, while the TUNL and 5-CSRTT data generally showed a decline with age, this trend was not universal; some 22-month-old mice performed on par with 12-month-old adults. These intriguing preliminary findings suggest that certain older individuals might not exhibit the typical cognitive performance decline, opening avenues to identify endophenotypes in aged mice with varying susceptibilities to cognitive decline. Investigating larger cohorts and expanding the approach to include genetic, neurohistochemical, or various omics analyses could be instrumental in identifying factors that respond to cognitive training, potentially enhancing cognition throughout the life span.

## Data availability statement

The raw data supporting the conclusions of this article will be made available by the authors, without undue reservation.

## Ethics statement

The animal study was approved by all procedures were conducted in compliance with the European Communities Council Directive 2010/63/EU and under the supervision and with the approval of the animal welfare officer at Humboldt University. The study was conducted in accordance with the local legislation and institutional requirements.

## Author contributions

DA: Writing – original draft, Conceptualization. AS: Software, Writing – review & editing. KS: Supervision, Writing – review & editing. YW: Supervision, Writing – review & editing, Conceptualization.
