# Supplementary material for: Cognitive training of mice attenuates age-related decline in associative learning and behavioral flexibility
Source: Front Behav Neurosci. 2024 Mar 14;18:1326501. doi: 10.3389/fnbeh.2024.1326501 (PMC10976437; doi:10.3389/fnbeh.2024.1326501)
Supplement: Supplementary file 1 [file Data_Sheet_1.docx]

**SUPPLEMENTARY INFORMATION**

**This document includes:**

**Supplement 1-6**

**Figures S1 to S10**

**Tables S1 to S7**

## Supplement:

1. **Apparatus**

The animal sorter consisted of a tube with two motor-controlled guillotine doors at each end and three RFID readers (RFID 1-3) to detect and identify mice. Detection at RFID 1 while sorter and operant chamber were unoccupied opened door one. Once the mouse was identified at RFID 3, door one closed and the animal remained within the sorter for 30 seconds. Detection by RFID readers two and three of only one mouse during this period resulted in the opening of door two allowing a single mouse to proceed to the operant chamber. Detection of more than one mouse in the sorter resulted in the opening of door one, thus re-starting the sorting procedure.

The operant unit consisted of a standard, trapezoidal touchscreen operant chamber with black Perspex walls, and a stainless-steel grid floor. A food magazine equipped with an infrared head entry detector was located opposite the touchscreen (1920 x 1200 pixels) and attached to a 14mg pellet dispenser. In front of the touchscreen was a black Perspex mask with either three or five response windows (5x5 cm, gap 1.9 cm or 6x3 cm, gap 1.4 cm, respectively). A grid of infrared photodetectors (IR) detected nose pokes toward the screen. This detector grid was placed behind the mask for cohort Y1, and in front of the mask for all other cohorts to improve nose poke detection. An additional 3.5 cm high barrier was placed in front of the IR grid to prevent unintentional touches for example by the tail of a mouse. For all cohorts except Y1 a divider equipped with a passive infrared detector (PIR) above a central opening was placed halfway between the touchscreen and the food magazine for the 5-CSRTT. This setup allowed animals to initiate trials only while travelling back from the food magazine toward the touchscreen ensuring that they were facing the touchscreen (Figure 1).

The chamber also had a loudspeaker and a house light, implemented by using the first top 220 pixel-rows of the screen.

### Data analysis

Generalized linear mixed models (GLMM) were used to model (a) trials to criterion and perseveration score as a function of training stage and age/prior experience during TUNL acquisition (b) trials to criterion as a function of stimulus duration and age/prior experience during 5-CSRTT acquisition (c) performance, omission rate, premature response rate, perseveration score as a function of stimulus duration and age/prior experience during 5-CSRTT testing, (d) trials to criterion as a function of phase and age/prior experience during visual discrimination and reversal learning phases. Age/ prior experience and spatial separation/ stimulus durations/ phase were added as fixed effects in the respective models. We first created intercept-only models and added random slopes by stage (spatial separation/stimulus durations/ phase) for fixed effects before interactions between random slopes were added.

Generalized linear regression was used to model (a) performance as a function of spatial separation and age/prior experience during TUNL spatial separation testing (b) performance as a function of delay and age/prior experience during TUNL delay testing. Random slope was kept if it improved the model according to the Akaike information criterion (AIC) (Myung, Forster, & Browne, 2000).

As session participation and performance partly depends on the mouse’s satiety state and thus eagerness to eat, sessions with 0% correct responses or less than five trials were not included in this analysis.

Three middle-aged mice failed to reach the criterion within a time frame comparable to the rest of the group but were still moved forward to the 5W learning stage. They were excluded from the trials to criterion analysis. However, since they successfully learned the task during 5W training (Figure S4), they were included in the probe trials analysis. For the 5-CSRTT acquisition analysis, two mice from cohort M12-mo were excluded as they failed to complete the training stage. Three mice were excluded from the 5-CSRTT testing phase analysis; one mouse was excluded from cohort Y2 4-mo as it was mistakenly given different testing conditions, and two mice from cohort O1 22-mo were excluded because of age-related reduced visual ability tested using a reflex-based behavioral task.

The perseveration score is given as the average number of correction trials per incorrect response.

## Figures

| 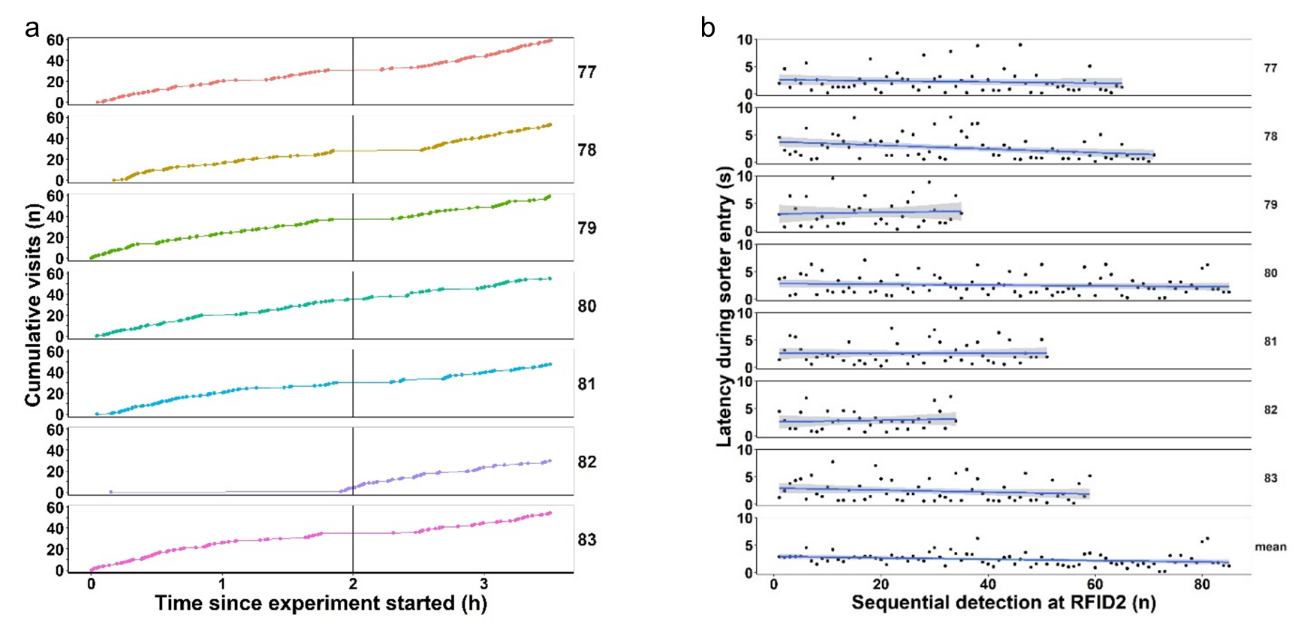 |
| --- |
| 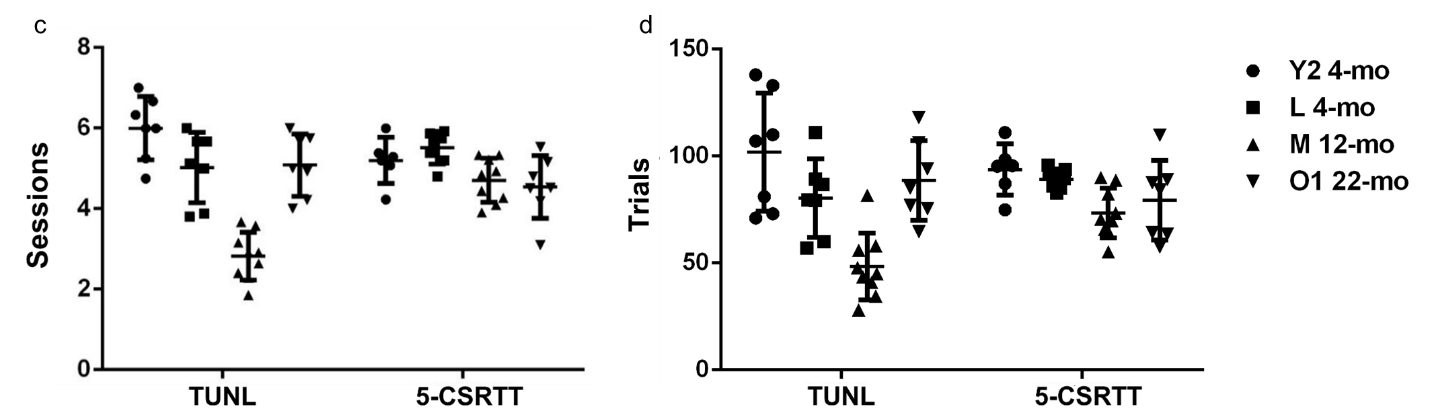 |

Figure S1. Sorter habituation and usage **(a)** Cumulative number of visits per individual (ID 77-83) during the first 3.5h of sorter exposure (exemplary data from cohort Y1). The vertical line indicates the start of the dark phase coinciding with the end of the sorter habituation phase **(b)** Latency of an animal (ID77-83) to be detected between reader 1 and 2 across visits during the first 3.5h. Blue lines represent linear regression best fits, and the grey bandwidth represents the 95% confidence interval. Latency decreased significantly across several visits but with small effect (adjusted R2 = 0.0227, F (5599, 457.4) =12.24, p<0.001). (Exemplary data from cohort Y1.) **(c)** Mean number of analysed sessions **(**trials >4 and % correct >0) per individual during TUNL and 5CSRTT tasks acquisition. Mice performed an average of 4.6 ± 0.26 individual sessions per day in the home-cage-based system. Symbols represent individual animals, lines with error bars represent cohort means ± SD. **(d)** Mean number of trials per individual during TUNL and 5CSRTT tasks acquisition. Mice performed an average of 82 ± 15.7 individual trials per day in the home-cage-based system. Symbols represent individual animals, lines with error bars represent cohort means ± SD.

| 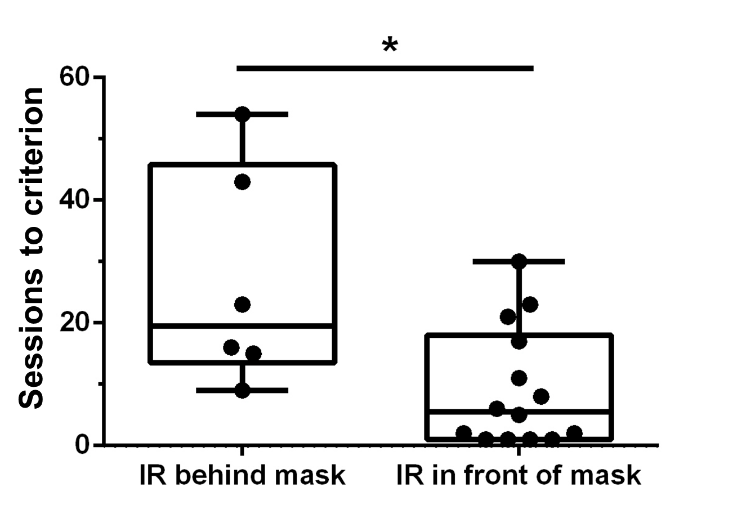  a | 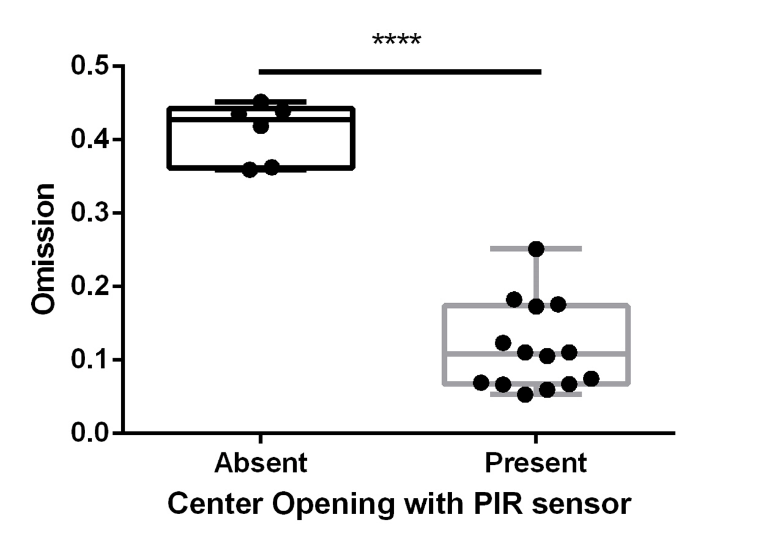  b |
| --- | --- |

Figure S2. Consequences of apparatus modifications. **(a)** Task acquisition (TUNL S3 spatial separation) with IR-nose poke detection behind or in front of the window mask. Placing the IR nose poke detection in front of the mask significantly reduced the number of sessions required to reach criterion. **(b)** Forcing animals to face the touchscreen at trial initiation by addition of a divider with central opening and PIR sensor detection significantly decreased omission rates in the 5-CSRTT. Symbols represent individual animals. Box plots show median, 25th–75th percentile, min- and maximum values. * p<0.05, **** p<0.0001

| 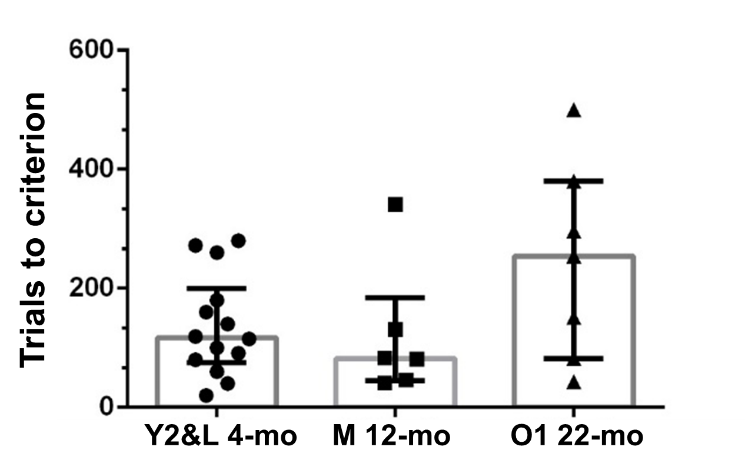 |
| --- |

Figure S3. Trials to reach criterion during TUNL-3W. No statistically significant age differences were observed during three window TUNL acquisition, although variance appeared higher in the O1 22-mo group. Bar graphs represent median ± SD. Symbols represent individual animals.


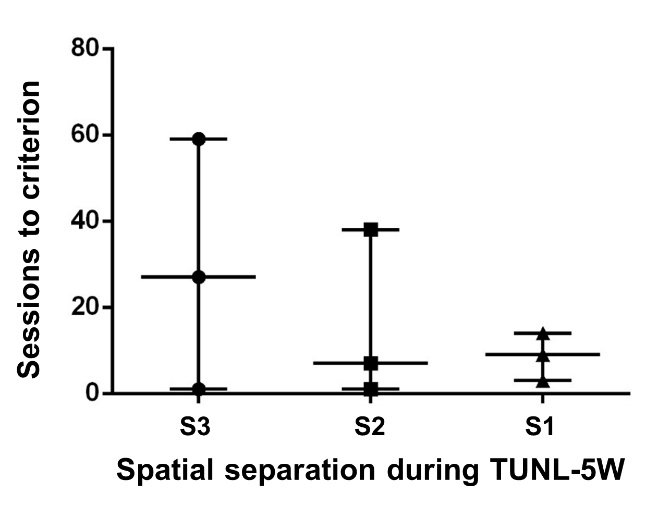


Figure S4. Three mice that failed to reach criterion during TUNL-3W, moved on to TUNL-5W and successfully acquired the task. Data are median and interquartile range.

## Tables

**Table S1.** Trials to criterion during 5 Window TUNL task acquisition. Poisson regression model with trials to criterion predicted by cohort and spatial separation (S3, S2, S1) as fixed effects, individuals as random-effect intercept and spatial separation as random-effect slope.

| *Predictors* | *Log-Mean (CI)* | *IRR (CI)* | *p* |
| --- | --- | --- | --- |
| (Intercept) | 3.80 ^***^ (3.26 – 4.34) | 44.69 ^***^ (26.17 – 76.34) | **<0.001** |
| S2 | -0.22  (-0.83 – 0.40) | 0.80 (0.44 – 1.49) | 0.486 |
| S1 | -0.26  (-0.96 – 0.43) | 0.77 (0.38 – 1.54) | 0.458 |
| M 12-mo | 1.01 ^***^ (0.71 – 1.30) | 2.74 ^***^(2.04 – 3.67) | **<0.001** |
| O1 22-mo | 0.46  (-0.56 – 1.49) | 1.59 (0.57 – 4.42) | 0.375 |
| S2 * M 12-mo | -0.49  (-1.52 – 0.53) | 0.61 (0.22 – 1.70) | 0.346 |
| S1 * M 12-mo | -0.21 (-0.83 – 0.40) | 0.81 (0.44 – 1.50) | 0.502 |
| S2 * O1 22-mo | 0.03 (-1.08 – 1.13) | 1.03 (0.34 – 3.09) | 0.964 |
| S1 * O1 22-mo | 0.03 (-1.28 – 1.34) | 1.03 (0.28 – 3.83) | 0.963 |

^S: spatial separation, M: middle-aged, O: old age, CI: confidence interval, IRR: incidence rate ratios; exponentiated model coefficient. Coefficient estimates and IRR with 95% credibility limits.^

**Table S2. Perseveration score during TUNL task acquisition.** Poisson regression model with perseveration score predicted by cohort and spatial separation (S3, S2, S1) as fixed effects, individuals as random-effect intercept, and spatial separation as random-effect slope.

| *Predictors* | *Log-Mean (CI)* | *IRR (CI)* | *p* |
| --- | --- | --- | --- |
| (Intercept) | -0.62 ^***^ (-0.85 – -0.40) | 0.54 ^***^ (0.43 – 0.67) | **<0.001** |
| S2 | 0.11 (-0.08 – 0.29) | 1.12 (0.93 – 1.34) | 0.249 |
| S1 | -0.15 (-0.35 – 0.04) | 0.86 (0.70 – 1.04) | 0.127 |
| M 12-mo | 0.66 ^***^ (0.41 – 0.91) | 1.94 ^***^ (1.51 – 2.49) | **<0.001** |
| O1 22-mo | 0.78 ^***^ (0.45 – 1.11) | 2.18 ^***^ (1.57 – 3.04) | **<0.001** |
| S2 * M 12-mo | -0.37 ^**^ (-0.61 – -0.14) | 0.69 ^**^ (0.54 – 0.87) | **0.002** |
| S1 * M 12-mo | -0.37 ^**^ (-0.63 – -0.12) | 0.69 ^**^ (0.53 – 0.89) | **0.004** |
| S2 * O1 22-mo | -0.05  (-0.30 – 0.20) | 0.95 (0.74 – 1.22) | 0.702 |
| S1 * O1 22-mo | -0.15 (-0.44 – 0.13) | 0.86 (0.64 – 1.14) | 0.294 |

^S: spatial separation, M: middle-aged, O: old age, CI: confidence interval, IRR: incidence rate ratios; exponentiated model coefficient. Coefficient estimates and IRR with 95% credibility limits.^

**Table S3.** Age effects during TUNL probe trials. Binominal regression model with proportion of correct responses predicted by cohort and spatial separation (S1 & S0, 0s delay).

| *Predictors* | *Log-Odds (CI)* | *OR (CI)* | *p* |
| --- | --- | --- | --- |
| (Intercept) | 1.06 ^***^ (0.91 – 1.22) | 2.88 ^***^ (2.48 – 3.37) | **<0.001** |
| S0 | -0.22 ^*^ (-0.43 – -0.01) | 0.81 ^*^ (0.65 – 0.99) | **0.044** |
| M 12-mo | -0.22 (-0.46 – 0.01) | 0.80 (0.63 – 1.01) | 0.059 |
| O1 22-mo | -0.25 ^*^ (-0.50 – 0.00) | 0.78 ^*^ (0.60 – 1.00) | **0.049** |
| S0 * M 12-mo | 0.11 (-0.22 – 0.43) | 1.11 (0.81 – 1.54) | 0.513 |
| S0 * O1 22-mo | 0.21 (-0.14 – 0.56) | 1.23 (0.87 – 1.75) | 0.242 |

^S: spatial separation, M: middle-aged, O: old age, CI: confidence interval, OR: odds ratios; exponentiated logits. Coefficient estimates and OR with 95% credibility limits.^

**Table S4.** Binominal regression model with proportion of correct responses predicted by cohort and delay (0, 3 & 6s delay; S1).

| *Predictors* | *Log-Odds (CI)* | *OR (CI)* | *p* |
| --- | --- | --- | --- |
| (Intercept) | 0.55 ^***^ (0.32 – 0.79) | 1.74 ^***^ (1.38 – 2.21) | **<0.001** |
| 3s Delay | -0.18 (-0.50 – 0.15) | 0.84 (0.61 – 1.16) | 0.284 |
| 6s Delay | -0.37 ^*^ (-0.69 – -0.05) | 0.69 ^*^ (0.50 – 0.95) | **0.024** |
| M 12-mo | -0.25 (-0.61 – 0.12) | 0.78 (0.54 – 1.12) | 0.179 |
| O1 22-mo | -0.41 ^*^ (-0.81 – -0.02) | 0.66 ^*^ (0.45 – 0.98) | **0.041** |
| 3s Delay * M 12-mo | -0.01 (-0.51 – 0.50) | 0.99 (0.60 – 1.64) | 0.977 |
| 3s Delay * O1 22-mo | 0.35 (-0.20 – 0.91) | 1.42 (0.82 – 2.49) | 0.214 |
| 6s Delay * M 12-mo | 0.28 (-0.22 – 0.78) | 1.32 (0.80 – 2.18) | 0.279 |
| 6s Delay * O1 22-mo | 0.31 (-0.22 – 0.84) | 1.36 (0.80 – 2.32) | 0.253 |

^S: spatial separation, M: middle-aged, O: old age, CI: confidence interval, OR: odds ratios; exponentiated logits. Coefficient estimates and OR with 95% credibility limits.^

**Table S5.** Binominal regression model with proportion of correct responses predicted by cohort and delay (0, 3 & 6s delay; S0).

| *Predictors* | *Log-Odds* | *OR* | *p* |
| --- | --- | --- | --- |
| (Intercept) | 0.59 ^***^ (0.37 – 0.81) | 1.80 ^***^ (1.44 – 2.26) | **<0.001** |
| 3s Delay | -0.33 ^*^ (-0.65 – -0.02) | 0.72 ^*^ (0.52 – 0.98) | **0.036** |
| 6s Delay | -0.53 ^***^ (-0.85 – -0.22) | 0.59 ^***^ (0.43 – 0.80) | **0.001** |
| M 12-mo | -0.45 ^*^ (-0.80 – -0.11) | 0.64 ^*^ (0.45 – 0.90) | **0.010** |
| O1 22-mo | -0.43 ^*^ (-0.80 – -0.06) | 0.65 ^*^ (0.45 – 0.94) | **0.024** |
| 3s Delay * M 12-mo | 0.35 (-0.14 – 0.84) | 1.42 (0.87 – 2.32) | 0.158 |
| 3s Delay * O1 22-mo | 0.44 (-0.06 – 0.94) | 1.55 (0.94 – 2.55) | 0.085 |
| 6s Delay * M 12-mo | 0.23 (-0.29 – 0.76) | 1.26 (0.75 – 2.13) | 0.385 |
| 6s Delay * O1 22-mo | 0.27 (-0.28 – 0.82) | 1.31 (0.76 – 2.26) | 0.335 |

^S: spatial separation, M: middle-aged, O: old age, CI: confidence interval, OR: odds ratios; exponentiated logits. Coefficient estimates and OR with 95% credibility limits.^

**Table S6.** Poisson regression model with trials to criteria predicted by cohort and stimulus durations (mean centred) as fixed effects, individuals as random-effect intercept and stimulus durations (mean centred) as random-effect slope.

| *Predictors* | *Log-Mean (CI)* | *IRR (CI)* | *p* |
| --- | --- | --- | --- |
| (Intercept) | 3.84 ^***^ (3.42 – 4.270) | 46.70 ^***^ (30.54 – 71.41) | **<0.001** |
| SD (scaled) | -0.70 ^**^ (-1.19 – -0.21) | 0.50 ^**^ (0.31 – 0.81) | **0.005** |
| M 12-mo | 1.64 ^***^ (1.40 – 1.88) | 5.16 ^***^ (4.05 – 6.57) | **<0.001** |
| O1 22-mo | 1.98 ^***^ (1.72 – 2.24) | 7.26 ^***^ (5.59 – 9.44) | **<0.001** |
| SD (scaled) * M 12-mo | 0.75 ^***^ (0.51 – 1.00) | 2.13 ^***^ (1.66 – 2.71) | **<0.001** |
| SD (scaled) * O1 22-mo | 0.68 ^***^ (0.40 – 0.96) | 1.98 ^***^ (1.50 – 2.62) | **<0.001** |

^SD: stimulus duration, M: middle-aged, O: old age, IRR: incidence rate ratios; exponentiated model coefficient. Coefficient estimates and IRR with 95% credibility limits.^

**Table S7.** Binominal regression model with proportion of correct responses predicted by cohort and stimulus durations (mean centred) as fixed effects, individuals as random-effect intercept, and stimulus durations (mean centred) as random-effect slope.

| *Predictors* | *Log-Odds (CI)* | *OR (CI)* | *p* |
| --- | --- | --- | --- |
| (Intercept) | 0.28 ^**^ (0.09 – 0.46) | 1.32 ^**^ (1.10 – 1.58) | **0.003** |
| SD (scaled) | 0.36 ^***^ (0.29 – 0.42) | 1.43 ^***^ (1.34 – 1.52) | **<0.001** |
| M 12-mo | -0.17 (-0.37 – 0.03) | 0.84 (0.69 – 1.03) | 0.101 |
| O1 22-mo | -0.47 ^*^ (-0.84 – -0.09) | 0.63 ^*^ (0.43 – 0.91) | **0.015** |
| SD (scaled) * M 12-mo | -0.01 (-0.12 – 0.10) | 0.99 (0.89 – 1.10) | 0.854 |
| SD (scaled) * O1 22-mo | 0.08 (-0.04 – 0.20) | 1.08 (0.96 – 1.22) | 0.213 |

^SD: stimulus duration, M: middle-aged, O: old age, CI: confidence interval, OR: odds ratios; exponentiated logits. Coefficient estimates and OR with 95% credibility limits.^

**Table S8.** Binominal regression model with proportion of no responses predicted by cohort and stimulus durations (mean centred) as fixed effects, individuals as random-effect intercept and stimulus durations (mean centred) as random-effect slope.

| *Predictors* | *Log-Odds (CI)* | *OR (CI)* | *p* |
| --- | --- | --- | --- |
| (Intercept) | -2.45 ^***^ (-2.73 – -2.18) | 0.09 ^***^ (0.07 – 0.11) | **<0.001** |
| SD (scaled) | -0.67 ^***^ (-0.81 – -0.52) | 0.51 ^***^ (0.44 – 0.59) | **<0.001** |
| M 12-mo | 1.24 ^***^ (0.93 – 1.55) | 3.45 ^***^ (2.53 – 4.71) | **<0.001** |
| O1 22-mo | 0.68 ^*^ (0.13 – 1.23) | 1.97 ^*^ (1.14 – 3.41) | **0.016** |
| SD (scaled) * M 12-mo | 0.15 (-0.08 – 0.39) | 1.17 (0.92 – 1.47) | 0.199 |
| SD (scaled) * O1 22-mo | 0.14 (-0.09 – 0.37) | 1.15 (0.91 – 1.45) | 0.240 |

^SD: stimulus duration, M: middle-aged, O: old age, CI: confidence interval, OR: Odds Ratios; exponentiated logits. Coefficient estimates and OR with 95% credibility limits.^

**Table S9.** Poisson regression model with the premature responses predicted by cohort and stimulus durations (mean centred) as fixed effects, individuals as random-effect intercept.

| *Predictors* | *Log-Mean (CI)* | *IRR (CI)* | *p* |
| --- | --- | --- | --- |
| (Intercept) | 3.12 ^***^ (2.80 – 3.45) | 22.74 ^***^  (16.49 – 31.37) | **<0.001** |
| SD (scaled) | 0.02 (-0.02 – 0.05) | 1.02 (0.98 – 1.05) | 0.335 |
| M 12-mo | 0.71 ^***^ (0.48 – 0.95) | 2.04 ^***^ (1.62 – 2.58) | **<0.001** |
| O1 22-mo | 0.99 ^**^ (0.31 – 1.68) | 2.70 ^**^ (1.36 – 5.35) | **0.004** |
| SD (scaled) * M 12-mo | 0.00 (-0.05 – 0.06) | 1.00 (0.95 – 1.06) | 0.867 |
| SD (scaled) * O1 22-mo | -0.01 (-0.06 – 0.04) | 0.99 (0.94 – 1.04) | 0.682 |

^SD: stimulus duration, M: middle-aged, O: old age, CI: confidence interval, IRR: incidence rate ratios; exponentiated model coefficient. Coefficient estimates and IRR with 95% credibility limits.^

**Table S10.** Poisson regression model with the trials to criterion predicted by cohort and spatial separation (S3, S2, S1) as fixed effects, individuals as random-effect intercept and spatial separation as random-effect slope.

| *Predictors* | *Log-Mean (CI)* | *IRR (CI)* | *p* |
| --- | --- | --- | --- |
| (Intercept) | 4.55 ^***^ (4.23 – 4.88) | 95.03 ^***^ (68.79 – 131.29) | **<0.001** |
| S2 | -0.38 ^***^ (-0.52 – -0.24) | 0.68 ^***^ (0.59 – 0.78) | **<0.001** |
| S1 | -0.66 ^***^ (-0.77 – -0.54) | 0.52 ^***^ (0.46 – 0.58) | **<0.001** |
| L 12-mo exp | -1.16 ^***^ (-1.41 – -0.91) | 0.31 ^***^ (0.24 – 0.40) | **<0.001** |
| S2 * L 12-mo exp | 0.22 (-0.03 – 0.48) | 1.25 (0.97 – 1.62) | 0.084 |
| S1 * L 12-mo exp | 0.92 ^***^ (0.70 – 1.15) | 2.52 ^***^ (2.02 – 3.15) | **<0.001** |

^S: spatial separation, L: longitudinal cohort, exp: prior experience at 4-mo, CI: confidence interval, IRR: incidence rate ratios; exponentiated model coefficient. Coefficient estimates and IRR with 95% credibility limits. Exp: cohort with prior experience.^

**Table S11.** Poisson regression model with the perseveration score by cohort and spatial separation (S3, S2, S1) as fixed effects, individuals as random-effect intercept and spatial separation as random-effect slope.

| *Predictors* | *Log-Mean (CI)* | *IRR (CI)* | *p* |
| --- | --- | --- | --- |
| (Intercept) | 0.05 (-0.16 – 0.26) | 1.05 (0.85 – 1.29) | 0.500 |
| S2 | -0.28 ^***^ (-0.43 – -0.14) | 0.75 ^***^ (0.65 – 0.87) | **<0.001** |
| S1 | -0.53 ^***^ (-0.69 – -0.38) | 0.59 ^***^ (0.50 – 0.69) | **<0.001** |
| L 12-mo exp | -0.68 ^**^ (-1.09 – -0.27) | 0.51 ^**^ (0.34 – 0.76) | **0.001** |
| S2 * L 12-mo exp | 0.23 (-0.32 – 0.79) | 1.26 (0.72 – 2.21) | 0.410 |
| S1 * L 12-mo exp | 0.46  (-0.04 – 0.95) | 1.58  (0.96 – 2.58) | 0.070 |

^S: spatial separation, L: longitudinal cohort, exp: prior experience at 4-mo, CI: confidence interval, IRR: incidence rate ratios; exponentiated model coefficient. Coefficient estimates and IRR with 95% credibility limits.^

**Table S12.** Binominal regression model with proportion of correct responses predicted by cohort and spatial separation (S1 & S0, 0s delay).

| *Predictors* | *Log-Odds (CI)* | *OR (CI)* | *p* |
| --- | --- | --- | --- |
| (Intercept) | 0.83 ^***^ (0.66 – 1.01) | 2.30 ^***^ (1.94 – 2.75) | **<0.001** |
| S0 | -0.11 (-0.35 – 0.14) | 0.90 (0.70 – 1.15) | 0.390 |
| L 12-mo exp | 0.29 ^*^ (0.01 – 0.58) | 1.34 ^*^ (1.01 – 1.78) | **0.043** |
| S0 * L 12-mo exp | -0.10 (-0.50 – 0.29) | 0.90 (0.61 – 1.33) | 0.604 |

^S: spatial separation, L: longitudinal cohort, exp: prior experience at 4-mo, CI: confidence interval, OR: Odds Ratios; exponentiated logits. Coefficient estimates and OR with 95% credibility limits.^

**Table S13.** Binominal regression model with proportion of correct responses predicted by cohort and delay (0, 3 & 6s delay; S1).

| *Predictors* | *Log-Odds (CI)* | *OR (CI)* | *p* |
| --- | --- | --- | --- |
| (Intercept) | 0.30 ^*^ (0.03 – 0.58) | 1.36 ^*^ (1.03 – 1.79) | **0.031** |
| 3s Delay | -0.18 (-0.57 – 0.20) | 0.83 (0.57 – 1.22) | 0.350 |
| 6s Delay | -0.09 (-0.48 – 0.29) | 0.91 (0.62 – 1.34) | 0.640 |
| L 12-mo exp | 0.57 ^*^ (0.11 – 1.04) | 1.77 ^*^ (1.12 – 2.82) | **0.015** |
| 3s Delay * L 12-mo exp | -0.47 (-1.10 – 0.14) | 0.62 (0.33 – 1.15) | 0.133 |
| 6s Delay * L 12-mo exp | -0.69 ^*^ (-1.31 – -0.09) | 0.50 ^*^ (0.27 – 0.91) | **0.025** |

^S: spatial separation, L: longitudinal cohort, exp: prior experience at 4-mo, CI: confidence interval, OR: Odds Ratios; exponentiated logits. Coefficient estimates and OR with 95% credibility limits.^

**Table S14.** Binominal regression model with proportion of correct responses predicted by cohort and delay (0,3 & 6s delay; S0).

| *Predictors* | *Log-Odds (CI)* | *OR (CI)* | *p* |
| --- | --- | --- | --- |
| (Intercept) | 0.13 (-0.13 – 0.40) | 1.14 (0.88 – 1.49) | 0.316 |
| 3s Delay | 0.02 (-0.36 – 0.40) | 1.02 (0.70 – 1.49) | 0.920 |
| 6s Delay | -0.09 (-0.48 – 0.29) | 0.91 (0.62 – 1.34) | 0.637 |
| L 12-mo exp | 0.55 ^*^ (0.12 – 0.98) | 1.73 ^*^ (1.13 – 2.67) | **0.012** |
| 3s Delay * L 12-mo exp | -0.56 (-1.15 – 0.03) | 0.57 (0.32 – 1.03) | 0.063 |
| 6s Delay * L 12-mo exp | -0.69 ^*^ (-1.29 – -0.09) | 0.50 ^*^ (0.28 – 0.91) | **0.024** |

^S: spatial separation, L: longitudinal cohort, exp: prior experience at 4-mo, CI: confidence interval, OR: Odds Ratios; exponentiated logits. Coefficient estimates and OR with 95% credibility limits.^

**Table S15.** Poisson regression model with the trials to criteria predicted by cohort and stimulus durations (mean centred) as fixed effects, individuals as random-effect intercept, and stimulus durations (mean centred) as random-effect slope.

| *Predictors* | *Log-Mean (CI)* | *IRR (CI)* | *p* |
| --- | --- | --- | --- |
| (Intercept) | 4.98 ^***^ (4.70 – 5.26) | 145.21 ^***^ (109.88 – 191.90) | **<0.001** |
| SD (scaled) | 0.21 (-0.23 – 0.66) | 1.24 (0.79 – 1.93) | 0.350 |
| L 12-mo exp | -1.61 ^***^ (-1.83 – -1.38) | 0.20 ^***^ (0.16 – 0.25) | **<0.001** |
| SD (scaled) * L 12-mo exp | -0.75 ^***^ (-0.99 – -0.51) | 0.47 ^***^ (0.37 – 0.60) | **<0.001** |

^SD: stimulus duration, L: longitudinal cohort, exp: prior experience at 4-mo, CI: confidence interval, IRR: incidence rate ratios; exponentiated model coefficient. Coefficient estimates and IRR with 95% credibility limits.^

**Table S16.** Binominal regression model with proportion of correct responses predicted by cohort and stimulus durations (mean centred) as fixed effects, individuals as random-effect intercept, and stimulus durations (mean centred) as random-effect slope.

| *Predictors* | *Log-Odds (CI)* | *OR (CI)* | *p* |
| --- | --- | --- | --- |
| (Intercept) | -0.12 (-0.32 – 0.07) | 0.89 (0.73 – 1.08) | 0.226 |
| SD (scaled) | 0.31 ^***^ (0.19 – 0.43) | 1.36 ^***^ (1.21 – 1.54) | **<0.001** |
| L 12-mo exp | 0.84 ^***^ (0.61 – 1.06) | 2.31 ^***^ (1.85 – 2.89) | **<0.001** |
| SD (scaled) * L 12-mo exp | 0.22 ^*^ (0.04 – 0.40) | 1.25 ^*^ (1.04 – 1.49) | **0.016** |

^SD: stimulus duration, L: Longitudinal cohort, exp: prior experience at 4-mo, CI: confidence interval, OR: Odds Ratios; exponentiated logits. Coefficient estimates and OR with 95% credibility limits.^

**Table S17.** Binominal regression model with proportion of no responses predicted by cohort and stimulus durations (mean centred) as fixed effects, individuals as random-effect intercept, and stimulus durations (mean centred) as random-effect slope.

| *Predictors* | *Log-Odds (CI)* | *OR (CI)* | *p* |
| --- | --- | --- | --- |
| (Intercept) | -1.53 ^***^ (-1.82 – -1.23) | 0.22 ^***^ (0.16 – 0.29) | **<0.001** |
| SD (scaled) | -0.53 ^***^ (-0.66 – -0.40) | 0.59 ^***^ (0.52 – 0.67) | **<0.001** |
| L 12-mo exp | -1.03 ^***^ (-1.31 – -0.75) | 0.36 ^***^ (0.27 – 0.47) | **<0.001** |
| SD (scaled) * L 12-mo exp | -0.48 ^***^ (-0.69 – -0.27) | 0.62 ^***^ (0.50 – 0.76) | **<0.001** |

^SD: stimulus duration, L: longitudinal cohort, exp: prior experience at 4-mo, CI: confidence interval, OR: Odds Ratios; exponentiated logits. Coefficient estimates and OR with 95% credibility limits.^

**Table S18.** Poisson regression model with the premature responses predicted by cohort and stimulus durations (mean centred) as fixed effects, individuals as random-effect intercept.

| *Predictors* | *Log-Mean (CI)* | *IRR (CI)* | *p* |
| --- | --- | --- | --- |
| (Intercept) | 3.57 ^***^ (3.34 – 3.80) | 35.55 ^***^ (28.28 – 44.68) | **<0.001** |
| SD (scaled) | 0.02 (-0.02 – 0.07) | 1.02 (0.98 – 1.07) | 0.328 |
| L 12-mo exp | -0.39 ^***^ (-0.59 – -0.20) | 0.67 ^***^ (0.55 – 0.82) | **<0.001** |
| SD (scaled) * L 12-mo exp | -0.01 (-0.08 – 0.06) | 0.99 (0.92 – 1.06) | 0.745 |

^SD: stimulus duration, L: longitudinal cohort, exp: prior experience at 4-mo, CI: confidence interval, IRR: incidence rate ratios; exponentiated model coefficient. Coefficient estimates and IRR with 95% credibility limits.^

**Table S19.** Poisson regression model with the trials to criteria predicted by cohort and stimulus durations (mean centred) as fixed effects, individuals as random-effect intercept and stimulus durations (mean centred) as random-effect slope.

| *Predictors* | *Log-Mean (CI)* | *IRR (CI)* | *p* |
| --- | --- | --- | --- |
| (Intercept) | 4.04 ^***^ (3.52 – 4.55) | 56.78 ^***^ (33.94 – 94.98) | **<0.001** |
| SD (scaled) | 0.39 (-0.05 – 0.83) | 1.48 (0.95 – 2.30) | 0.083 |
| L 12-mo exp | -0.87 ^***^ (-0.95 – -0.79) | 0.42 ^***^ (0.38 – 0.45) | **<0.001** |
| SD (scaled) * L 12-mo exp | -0.42 ^***^ (-0.51 – -0.33) | 0.66 ^***^ (0.60 – 0.72) | **<0.001** |

^SD: stimulus duration, L: longitudinal cohort, exp: prior experience at 4-mo, CI: confidence interval, IRR: incidence rate ratios; exponentiated model coefficient. Coefficient estimates and IRR with 95% credibility limits.^

**Table S20.** Binominal regression model with proportion of correct responses predicted by cohort and stimulus durations (mean centred) as fixed effects, individuals as random-effect intercept and stimulus durations (mean centred) as random-effect slope.

| *Predictors* | *Log-Odds (CI)* | *OR (CI)* | *p* |
| --- | --- | --- | --- |
| (Intercept) | 0.19 (-0.01 – 0.39) | 1.21 (0.99 – 1.48) | 0.068 |
| SD (scaled) | 0.40 ^***^ (0.28 – 0.53) | 1.49 ^***^ (1.32 – 1.69) | **<0.001** |
| L 12-mo exp | 0.43 ^***^ (0.33 – 0.52) | 1.53 ^***^ (1.39 – 1.69) | **<0.001** |
| SD (scaled) * L 12-mo exp | 0.09 (-0.01 – 0.20) | 1.10 (0.99 – 1.22) | 0.080 |

^SD: stimulus duration, L: longitudinal cohort, exp: prior experience at 4-mo, CI: confidence interval, OR: Odds Ratios; exponentiated logits. Coefficient estimates and OR with 95% credibility limits.^

**Table S21.** Binominal regression model with proportion of no responses prediced by cohort and stimulus durations (mean centred) as fixed effects, individuals as random-effect intercept and stimulus durations (mean centred) as random-effect slope.

| *Predictors* | *Log-Odds (CI)* | *OR (CI)* | *p* |
| --- | --- | --- | --- |
| (Intercept) | -2.43 ^***^ (-2.73 – -2.13) | 0.09 ^***^ (0.07 – 0.12) | **<0.001** |
| SD (scaled) | -0.48 ^***^ (-0.66 – -0.31) | 0.62 ^***^ (0.52 – 0.74) | **<0.001** |
| L 12-mo exp | 0.12 (-0.04 – 0.28) | 1.13 (0.96 – 1.33) | 0.149 |
| SD (scaled) * L 12-mo exp | -0.44 ^***^ (-0.65 – -0.24) | 0.64 ^***^ (0.52 – 0.79) | **<0.001** |

^SD: stimulus duration, L: longitudinal cohort, exp: prior experience at 4-mo, CI: confidence interval, OR: Odds Ratios; exponentiated logits. Coefficient estimates and OR with 95% credibility limits.^

**Table S22.** Poisson regression model with the premature responses predicted by cohort and stimulus durations (mean centred) as fixed effects, individuals as random-effect intercept.

| *Predictors* | *Log-Mean (CI)* | *IRR (CI)* | *p* |
| --- | --- | --- | --- |
| (Intercept) | 2.85 ^***^ (2.58 – 3.12) | 17.35 ^***^ (13.23 – 22.74) | **<0.001** |
| SD (scaled) | 0.02 (-0.05 – 0.09) | 1.02 (0.96 – 1.10) | 0.492 |
| L 12-mo exp | 0.26 ^***^ (0.17 – 0.36) | 1.30 ^***^ (1.18 – 1.43) | **<0.001** |
| SD (scaled) * L 12-mo exp | -0.01 (-0.11 – 0.08) | 0.99 (0.90 – 1.08) | 0.753 |

^SD: stimulus duration, L: longitudinal cohort, exp: prior experience at 4-mo, CI: confidence interval, IRR: incidence rate ratios; exponentiated model coefficient. Coefficient estimates and IRR with 95% credibility limits.^

**Table S23.** Pairwise comparisons of estimated marginal means from the Poisson regression model.

| Cohort | Condition | Estimate | asymp.LCL | asymp.UCL | P value |
| --- | --- | --- | --- | --- | --- |
| L 22-mo exp – Y3 4-mo naive | VD | -0.62 | -0.84 | -0.40 | <.0001 |
| O2 22-mo naive – Y3 4-mo naive | VD | 1.20 | 0.67 | 1.74 | <.0001 |
| O2 22-mo naive – L 22-mo exp | VD | 1.82 | 1.28 | 2.37 | <.0001 |
| L 22-mo exp - Y3 4-mo naive | Reversal | -0.77 | -0.95 | -0.58 | <.0001 |
| O2 22-mo naive - Y3 4-mo naive | Reversal | 1.19 | 0.83 | 1.55 | <.0001 |
| O2 22-mo naive - Y3 22-mo exp | Reversal | 1.96 | 1.58 | 2.33 | <.0001 |

^Results are on the log scale. Confidence level used: 0.95. Trials to criterion are predicted by cohort and phases (Visual discrimination and reversal learning) as fixed effects, individuals as random-effect intercept and phases as random-effect slope. VD: visual discrimination, LCL: lower confidence level, UCL: upper confidence level, exp: prior experience with touchscreen system, L: longitudinal cohort, O: old-age, Y: Young.^

**Table S24**. TUNL task training: Comparison between studies with young-aged mice (3-4 months) in home-cage-based and conventional touchscreen chambers.

| Feature | Home-cage-based with sorter | Conventional | | |
| --- | --- | --- | --- | --- |
| Housing condition | Group | Group | Group | Group |
| Transfer to test chamber | Self-initiated, no handling | Handled by experimenter | Handled by experimenter | Handled by experimenter |
| Food deprivation | - | ✓ | ✓ | ✓ |
| Mask | 5x1 | 5x1 | 5x1 | 6x1 |
| Session length | 18 trials or 15 min | 48 trials or 60 min | 48 trials or 60 min | 48 trials or 60 min |
| Visits per day | 5 ± 0.9 (mean ± sd) visits | 1x | 1x | 1x |
| Criterion | two consecutive sessions with ≥ 70% correct responses | ≥70 % correct over two sessions | two consecutive sessions with ≥ 70% correct responses | two consecutive sessions of > 70% correct responses |
| Pre-training TUNL | 3 Window pre-training | No pre-training | No pre-training | Cued TUNL training |
| Days to criterion TUNL  pre-training (mean ± sd) | 4.8 ± 2.1 | - | - | 4.2 ± 0.74 |
| Days to criterion  S4/S3-S1 training (mean ± sd) | 7 ± 4.8 (S3-S1) | 13.3 ± 0.68 (S3-S1) | 20.5 ± 8.9 (S3-S1) | 15 ± 1.5 (S4-S1) |
| References | This study | (37) | (36) | (38) |

**Table S25**. 5-CSRTT training: Comparison between studies with young-aged mice (3-4 months) in home-cage-based and conventional operant chambers.

| Feature | Home cage-based with sorter | CombiCage | Conventional 5-hole operant  chamber | Conventional  touchscreen chamber | Conventional  touchscreen chamber |
| --- | --- | --- | --- | --- | --- |
| Housing condition | Group | Single | Group | Group | Group |
| Transfer to test area | Self-initiated, no handling | Self-initiated, no handling | Handled by experimenter | Handled by experimenter | Handled by experimenter |
| Food deprivation | - | - | ✓ | ✓ | ✓ |
| Animals prior experience | TUNL task | - | - | - | - |
| Session length | 20 trials or 15 min | 24-h/day | 60 trials or 25 min | 50 trials or 60 min | 50 trials in 60 min |
| Visits per day | 5 ± 0.5 (mean ± sd) visits | Free access | 1x | 1x | 1x |
| Criterion | two consecutive sessions with ≥ 70% correct responses and < 30% omissions | started trials >50, >60% correct responses and omissions <30% or number of correct responses > = 200 | started trials >50, >60% correct responses and omissions <30% | two consecutive sessions with >80% correct responses and <20% omissions | completing 50 trials at ≥80% correct responses and < 20% omissions in 60 minutes |
| Days to criterion (mean ± sd) | 12 ± 2.7 | 4.8 ± 1 | 51.5 ± 13 | N/A | N/A |
| Days to criterion at 2sec training stage | 4.1 ± 2.9 | N/A | N/A | 11.4 ± 0.9 | N/A |
| Days to criterion at 4sec training stage | 1.6 ± 0.5 | N/A | N/A | N/A | 8.17 ± 0.80 |
| References | This study | (27) | (27) | (35) | (43) |

*N/A: data not available*
